# Intracellular adenosine regulates epigenetic programming in endothelial cells to promote angiogenesis

**DOI:** 10.15252/emmm.201607066

**Published:** 2017-07-27

**Authors:** Yiming Xu, Yong Wang, Siyuan Yan, Yaqi Zhou, Qiuhua Yang, Yue Pan, Xianqiu Zeng, Xiaofei An, Zhiping Liu, Lina Wang, Jiean Xu, Yapeng Cao, David J Fulton, Neal L Weintraub, Zsolt Bagi, Md Nasrul Hoda, Xiaoling Wang, Qinkai Li, Mei Hong, Xuejun Jiang, Detlev Boison, Christian Weber, Chaodong Wu, Yuqing Huo

**Affiliations:** ^1^ Vascular Biology Center Department of Cellular Biology and Anatomy Medical College of Georgia Augusta University Augusta GA USA; ^2^ School of Basic Medical Sciences Guangzhou Medical University Guangzhou China; ^3^ College of Basic Medicine Chengdu University of Traditional Chinese Medicine Chengdu China; ^4^ State Key Laboratory of Mycology Institute of Microbiology Chinese Academy of Science Beijing China; ^5^ Drug Discovery Center Key Laboratory of Chemical Genomics Peking University Shenzhen Graduate School Shenzhen China; ^6^ Georgia Prevention Institute Augusta University Augusta GA USA; ^7^ Departments of Medical Laboratory, Imaging & Radiologic Sciences, and Neurology Augusta University Augusta GA USA; ^8^ Robert S. Dow Neurobiology Laboratories Legacy Research Institute Portland OR USA; ^9^ Institute for Cardiovascular Prevention Ludwig‐Maximilians‐University Munich Munich Germany; ^10^ Department of Nutrition and Food Science Texas A&M University College Station TX USA

**Keywords:** adenosine, adenosine kinase, angiogenesis, DNA methylation, endothelial cells, Chromatin, Epigenetics, Genomics & Functional Genomics, Vascular Biology & Angiogenesis

## Abstract

The nucleoside adenosine is a potent regulator of vascular homeostasis, but it remains unclear how expression or function of the adenosine‐metabolizing enzyme adenosine kinase (ADK) and the intracellular adenosine levels influence angiogenesis. We show here that hypoxia lowered the expression of ADK and increased the levels of intracellular adenosine in human endothelial cells. Knockdown (KD) of ADK elevated intracellular adenosine, promoted proliferation, migration, and angiogenic sprouting in human endothelial cells. Additionally, mice deficient in endothelial ADK displayed increased angiogenesis as evidenced by the rapid development of the retinal and hindbrain vasculature, increased healing of skin wounds, and prompt recovery of arterial blood flow in the ischemic hindlimb. Mechanistically, hypomethylation of the promoters of a series of pro‐angiogenic genes, especially for VEGFR2 in ADK KD cells, was demonstrated by the Infinium methylation assay. Methylation‐specific PCR, bisulfite sequencing, and methylated DNA immunoprecipitation further confirmed hypomethylation in the promoter region of VEGFR2 in ADK‐deficient endothelial cells. Accordingly, loss or inactivation of ADK increased VEGFR2 expression and signaling in endothelial cells. Based on these findings, we propose that ADK downregulation‐induced elevation of intracellular adenosine levels in endothelial cells in the setting of hypoxia is one of the crucial intrinsic mechanisms that promote angiogenesis.

## Introduction

Adenosine is known for its regulatory effects on vascular function (Drury & Szent‐Gyorgyi, 1929), including induction of endothelial cell proliferation and migration *in vitro* and vascular growth *in vivo* (Adair, 2005). However, changes in adenosine metabolism and the correlation of these changes with angiogenesis are not clearly understood. While much emphasis has been placed on the necessity of extracellular adenosine and adenosine receptors for the angiogenic effect of adenosine (Adair, 2005), the role of intracellular adenosine in angiogenesis has never been investigated.

**Figure 1:**
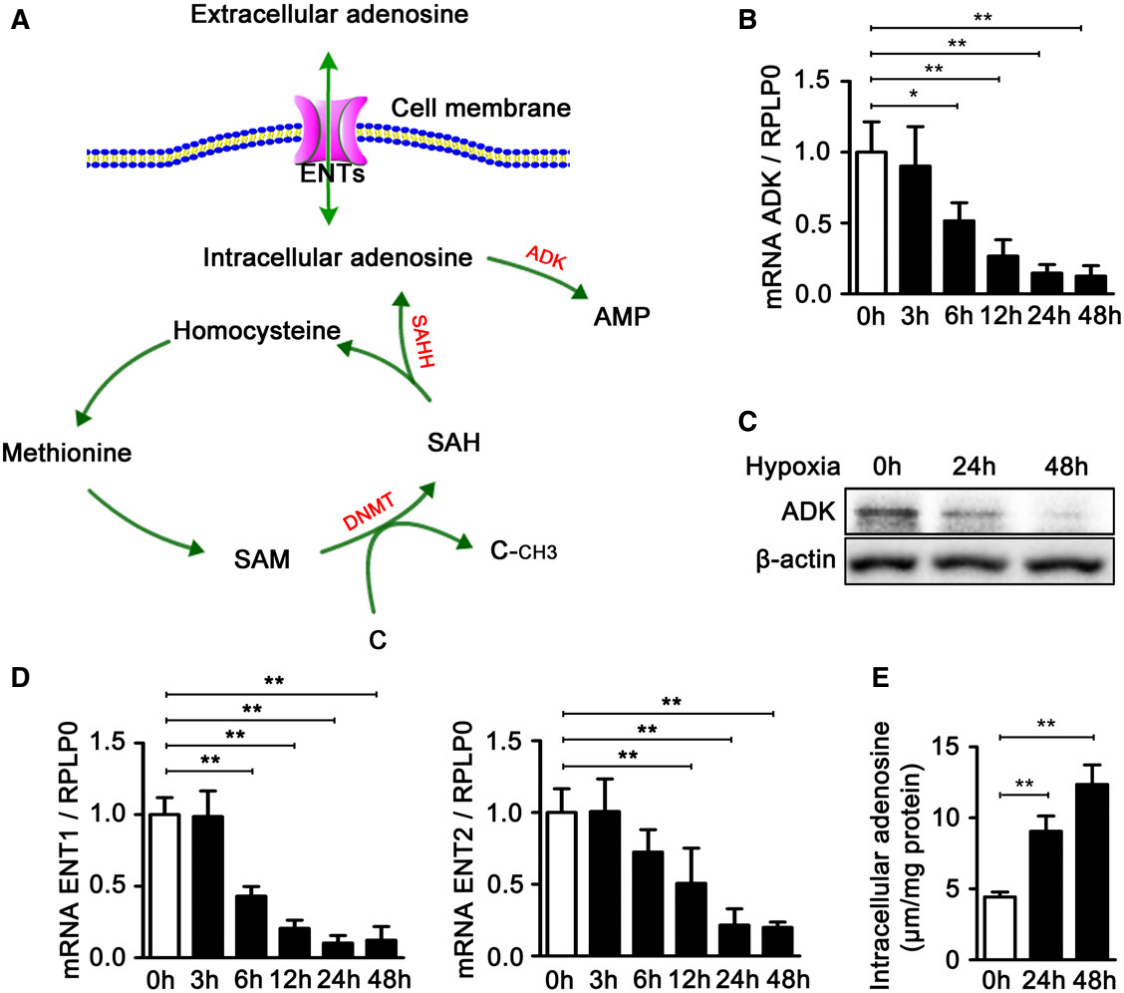
Decreased ADK expression and increased level of intracellular adenosine in endothelial cells upon hypoxia Biochemistry of the transmethylation reaction.Real‐time PCR analysis of ADK mRNA levels in HUVECs exposed to hypoxia for the indicated time periods. Results are from four independent experiments.Western blot analysis of ADK expression in HUVECs exposed to hypoxia for the indicated time periods. Images are representative from three independent experiments.Real‐time PCR analysis of ENT1 and ENT2 mRNA levels in HUVECs exposed to hypoxia for the indicated time periods. Results are from four independent experiments.Quantification of intracellular adenosine by high‐performance liquid chromatography (HPLC) in HUVECs exposed to hypoxia for the indicated time periods. Results are from four independent experiments.Data information: For all bar graphs, data are the mean ± SD, *P <* *0.05 and **P <* *0.01 for the indicated comparisons; one‐way ANOVA with Tukey's *post‐hoc* test. The exact *P*‐values are specified in Appendix Table S1. Source data are available online for this figure.

Intracellular adenosine is generated by stepwise dephosphorylation of adenosine triphosphate (ATP) or by the hydrolysis of *S*‐adenosylhomocysteine (SAH; Boison, 2013). *S*‐adenosylmethionine (SAM)‐dependent transmethylation generates SAH, which is then further converted into adenosine and homocysteine by SAH hydrolase (SAHH; Boison, 2013; Fig 1A). Since the equilibrium constant of SAHH enzyme lies in the direction of SAH formation, the transmethylation reactions will only continue when adenosine and homocysteine are constantly removed (Boison, 2013). Elevated adenosine regulates DNA methylation through interference with the transmethylation pathway (Williams‐Karnesky *et al*, 2013). However, it remains unknown whether adenosine affects angiogenesis via a DNA methylation‐dependent manner. Adenosine formed extracellularly can be transported into cells by means of efficient equilibrative nucleoside transporters (ENTs; Loffler *et al*, 2007). ENT inhibitors block the entrance of extracellular adenosine into cells when breakdown of extracellular adenine nucleotides results in production of a large amount of adenosine, and they also prevent outward transport of intracellular adenosine when a large amount of adenosine is formed intracellularly (Fredholm, 2007). Dipyridamole, which affects the intracellular adenosine level as an ENT inhibitor, has a paradoxical effect on endothelial healing and angiogenesis (Bomberger *et al*, 1982; Liem *et al*, 2001; Pattillo *et al*, 2011). Thus, it is necessary and urgent to elucidate the role of intracellular adenosine in angiogenesis.

Intracellular adenosine can be removed by adenosine deaminase (ADA) or by adenosine kinase (ADK). However, because of its much lower Michaelis constant (Drabikowska *et al*, 1985), ADK is regarded as the principal enzyme regulating intracellular adenosine concentrations under physiological conditions (Boison, 2013). In this study, we targeted ADK to study the angiogenic effect of intracellular adenosine and the involvement of DNA methylation in this action. We found that ADK deficiency increased intracellular adenosine level and promoted DNA hypomethylation in the promoter regions of some pro‐angiogenic genes. Epigenetic upregulation of these pro‐angiogenic genes by ADK deficiency promoted endothelial proliferation and migration *in vitro* and improved wound healing as well as hindlimb ischemia‐induced angiogenesis *in vivo*.

## Results

### Hypoxia increases endothelial intracellular adenosine through HIF‐1α‐dependent ADK downregulation *in vitro*

To elucidate the effect of hypoxia on the metabolism of endothelial adenosine, human umbilical vein endothelial cells (HUVECs) were cultured either in normoxia or hypoxia for different periods of time (0–48 h), and the expression of ADK, ENT1, and ENT2 was examined. As shown in Fig 1B and D, the mRNA levels of ADK, ENT1, and ENT2 were significantly decreased by hypoxia in a time‐dependent manner. Furthermore, the protein level of ADK was also significantly decreased in HUVECs upon hypoxia (Fig 1C). Hypoxia‐inducible factors (HIFs), particularly HIF‐1α and HIF‐2α, regulate the expression of substantive hypoxia‐response genes. We then examined whether HIFs were involved in ADK repression by hypoxia. To this end, as a first step, we performed loss‐of‐function studies utilizing small interfering RNAs (siRNAs) targeting HIF‐1α and HIF‐2α (Appendix Fig S1A and B). Knockdown (KD) of HIF‐1α almost fully abrogated the repression of ADK by hypoxia (Appendix Fig S1C), and KD of HIF‐2α, albeit partially, also significantly reversed the ADK downregulation in HUVECs exposed to hypoxia (Appendix Fig S1D). In addition, we carried out HIF‐1α or HIF‐2α gain‐of‐function studies using adenoviral vectors encoding transcriptionally active mutants of HIF‐1α (Ad‐mutHIF‐1α) or HIF‐2α (Ad‐mutHIF‐2α; Appendix Fig S1E and F). We found that overexpression of either HIF‐1α or HIF‐2α in HUVECs was associated with repression of ADK transcription under normoxic conditions (Appendix Fig S1G). Altogether, these findings show that hypoxia induces ADK repression via HIF‐α‐dependent mechanisms in endothelial cells.

Since ADK mediates the metabolism of intracellular adenosine and ENT mediates the transmembrane transport of adenosine, the downregulation of ADK, ENT1, and ENT2 by hypoxia prompted us to measure the level of intracellular adenosine. As shown in Fig 1E, the intracellular adenosine level was significantly elevated in HUVECs upon hypoxia, which might result from decreased clearance through ADK and decreased outward transport through ENT1 and ENT2.

### ADK KD in endothelial cells increases angiogenesis *in vitro* or *ex vivo*

**Figure 2:**
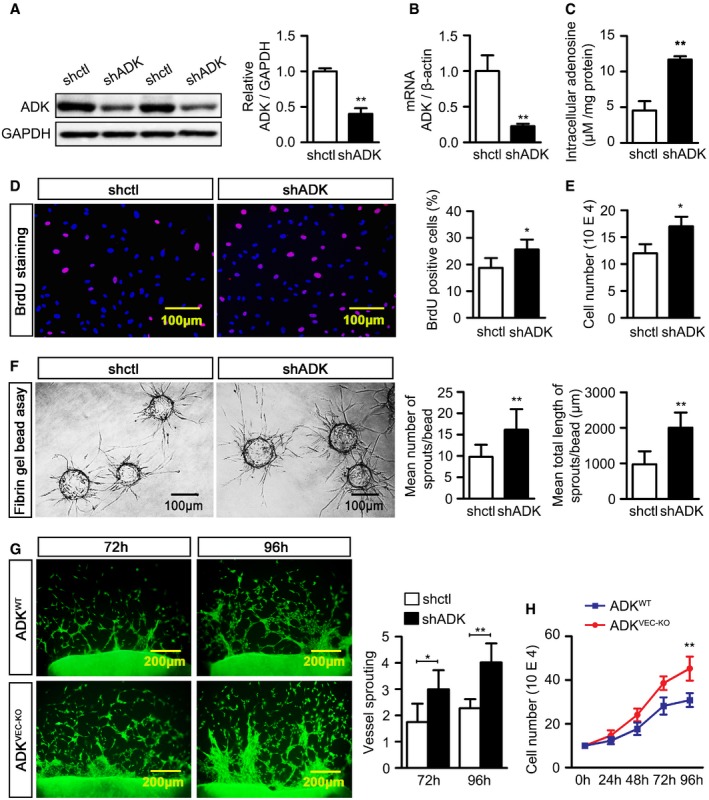
*In vitro* angiogenic activity of ADK deficiency in endothelial cells Western blot analysis of ADK expression in control (shctl) and ADK KD (shADK) HUVECs. Results are from three independent experiments.Real‐time PCR analysis of ADK mRNA levels in shctl and shADK HUVECs. Results are from three independent experiments.Quantification of intracellular adenosine in HUVECs by HPLC. Results are from three independent experiments.BrdU staining of proliferating HUVECs. Results are from four independent experiments.Cell numbers of HUVECs grown for 24 h. Results are from three independent experiments.Representative images of sprouting assay with fibrin gel and quantification of sprout numbers and sprout length (*n* = 30 beads per group).Representative images and quantification of endothelial cell outgrowth on days 3 and 4 after murine aortic rings were placed on collagen gel. Results are obtained from eight aortic rings isolated from four mice for each group.Number of MAECs from ADK^WT^ and ADK^VEC‐KD^ mice grown in complete growth medium for 0, 24, 48, 72, and 96 h. Results are from four independent experiments.Data information: For all bar graphs, data are the mean ± SD, *P <* *0.05 and **P <* *0.01 for the indicated comparisons; unpaired, two‐tailed Student's *t*‐test for (A–F); two‐way ANOVA with Bonferroni's *post‐hoc* test for (G and H). The exact *P*‐values are specified in Appendix Table S1. Source data are available online for this figure.

To investigate the role of ADK downregulation and the elevated level of intracellular adenosine in angiogenesis in endothelial cells, ADK was knocked down by an adenovirus encoding ADK shRNA, which dramatically decreased the ADK protein (Fig 2A) and mRNA levels (Fig 2B) and elevated the intracellular adenosine level over twofold (Fig 2C). Bromodeoxyuridine (BrdU) incorporation assay showed that the proliferation of endothelial cells was 32% higher in ADK KD HUVECs than in control cells (Fig 2D). This is consistent with a 41% increase in the number of ADK KD HUVECs within a growth period of 24 h compared with controls (Fig 2E). In a transwell assay, the number of ADK KD HUVECs migrating to the cell medium supplemented with VEGF at 50 ng/ml was 60% higher than that of control cells (Appendix Fig S2A). Capillary network formation on Matrigel was enhanced by 49% in ADK KD HUVECs compared with that of controls (Appendix Fig S2B). To further determine the role of ADK in the endothelial cell angiogenic response, we performed the three‐dimensional (3D) fibrin gel bead assay that recapitulates the various steps of angiogenesis, including sprouting, lumen formation, branching, and anastomosis (Nakatsu & Hughes, 2008; Morin *et al*, 2015). Quantitative analysis indicated that the number of sprouts and the total sprout length were drastically increased by ADK KD (Fig 2F). This was further confirmed in another 3D *in vitro* endothelial cell spheroid assay (Appendix Fig S2C), in which HUVEC spheroids were embedded in a collagen matrix (Simons *et al*, 2015).

**Figure 3:**
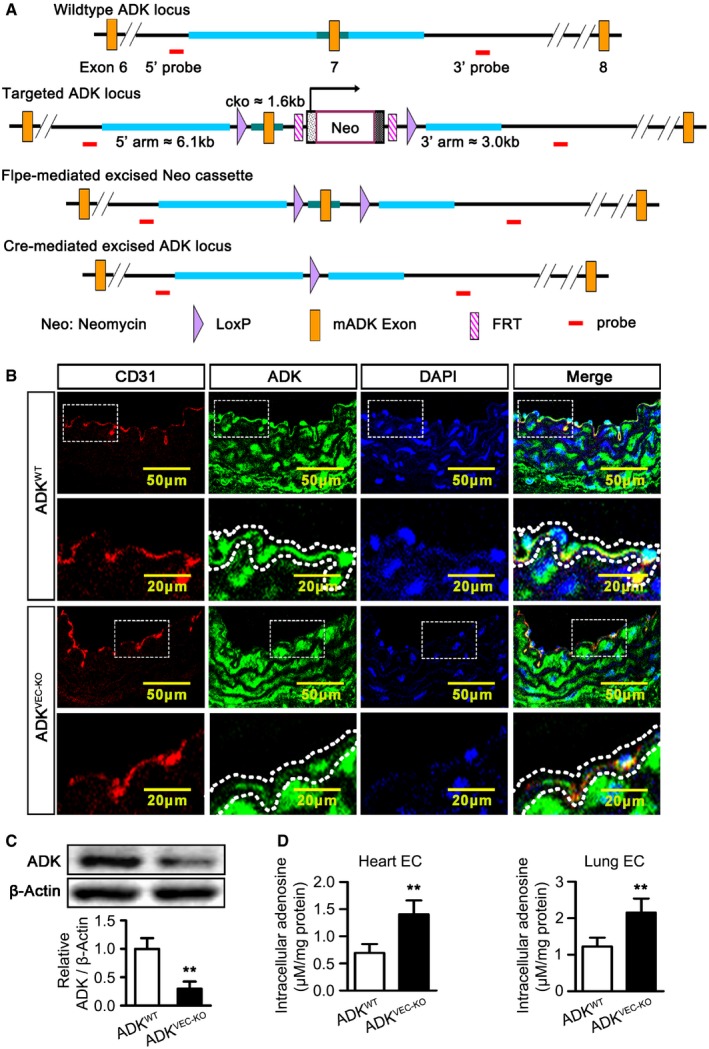
Generation and characterization of ADK^WT^ and ADK^VEC‐KO^ mice LoxP targeting of ADK. The targeting construct introduces the loxP sites flanking exon 7 of the ADK gene. Floxed mice were then crossed with a mouse in which expression of Cre recombinase is driven by an endothelial‐specific promoter associated with the vascular endothelial cadherin (Cdh5).Immunofluorescence staining of CD31 and ADK on aortic endothelium of ADK^WT^, Cdh5‐Cre (ADK^VEC‐KO^), and ADK^WT^ mice (*n* = 4 mice per group). Dotted lines outline the arterial endothelium.Western blot analysis of ADK in MAECs isolated from ADK^WT^ and ADK^VEC‐KO^ mice. Results are from four independent experiments.Quantification of intracellular adenosine by HPLC in mouse endothelial cells isolated from hearts and lungs. Results are from four independent experiments.Data information: For all bar graphs, data are the means ± SD, **P <* *0.01 for ADK^VEC‐KO^ vs. ADK^WT^; unpaired, two‐tailed Student's *t*‐test. The exact *P*‐values are specified in Appendix Table S1. Source data are available online for this figure.

To investigate the influence of ADK deficiency on angiogenesis *in vivo* or *ex vivo*, ADK^VEC‐KO^ mice with selective ADK deficiency in endothelial cells were generated via a tissue‐specific excision of the ADK gene using Cdh5‐Cre‐Lox technology (Fig 3A). Expression of ADK was significantly reduced in aortic endothelium in ADK^VEC‐KO^ mice (Fig 3B) and in mouse aortic endothelial cells (MAECs) isolated from ADK^VEC‐KO^ mice (Fig 3C). Additionally, the levels of intracellular adenosine in MAECs (data not shown), mouse lung microvascular endothelial cells, and mouse coronary microvascular endothelial cells isolated from ADK^VEC‐KO^ mice were significantly higher than those in cells isolated from wild‐type (WT) control mice (Fig 3D). In an *ex vivo* aortic ring assay, the average length and branch number of endothelial sprouts were consistently increased in aortas from ADK^VEC‐KO^ mice compared with those from littermate control ADK^WT^ mice (Fig 2G). Growth of MAECs was significantly increased for cells from ADK^VEC‐KO^ mice compared with those from ADK^WT^ mice (Fig 2H).

### Endothelial ADK deletion enhances angiogenesis *in vivo*

**Figure 4:**
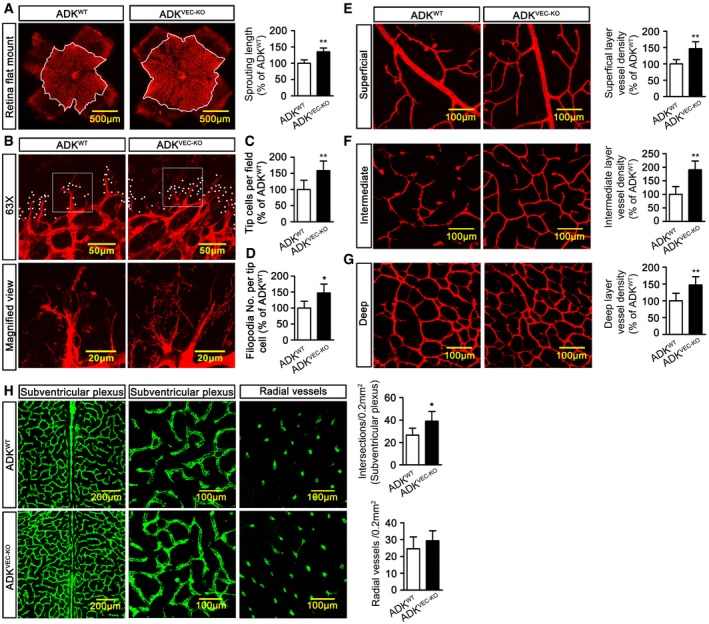
Retinal and hindbrain vascular development in mice with endothelial ADK deficiency ARepresentative images and quantification of isolectin B4‐stained whole‐mounted murine P4 retinas. White outlines indicate the range of vessel extension (*n* = 6 mice per group).BRepresentative images of filopodia projections from vascular sprouts in murine retinas. White circles indicate filopodia extended from tip cells.C, DQuantification of tip cells and filopodia of murine P4 retinas (*n* = 6 mice per group).ERepresentative images and quantification of vascular plexus in superficial layer of murine P12 retinas (*n* = 6 mice per group).FRepresentative images and quantification of vascular plexus in intermediate layer of murine P12 retinas (*n* = 6 mice per group).GRepresentative images and quantification of vascular plexus in deep layer of murine P12 retinas (*n* = 6 mice per group).HRepresentative images and quantification of vascular plexus in subventricular and radial layers in flat‐mount preparations of E11.5 hind brains (*n* = 6–8 mice per group).Data information: For all bar graphs, data are the means ± SD, *P <* *0.05, **P <* *0.01 for ADK^VEC‐KD^ vs. ADK^WT^; unpaired, two‐tailed Student's *t*‐test. The exact *P*‐values are specified in Appendix Table S1.

Previous studies have shown that hypoxia downregulates ADK and increases intracellular adenosine *in vivo* (Decking *et al*, 1997; Morote‐Garcia *et al*, 2008). To determine whether endothelial ADK deficiency modulates angiogenesis *in vivo*, development of the retinal vasculature was examined in 4‐day‐old pups of ADK^VEC‐KO^ mice and littermate controls. The spread of the retinal vasculature in ADK^VEC‐KO^ mice was 35% greater than in littermate controls (Fig 4A). The number of tip cells (Fig 4B and C) and filopodia (Fig 4B and D) were also markedly increased in ADK^VEC‐KO^ mice compared to littermate controls. In retinas from mice on postnatal day 12 (P12), the vascular plexus in the superficial (Fig 4E), intermediate (Fig 4F), and deep (Fig 4G) layers were markedly increased in ADK^VEC‐KO^ mice. We also analyzed the role of ADK in vessel formation in the hindbrain, another model utilized to study angiogenesis (Fantin *et al*, 2013). Histological analysis of flat‐mount preparations of hindbrains from ADK^VEC‐KO^ mice on embryonic day 11.5 (E11.5) revealed a higher number of vessel intersections and increased subventricular plexus complexity (Fig 4H).

**Figure 5:**
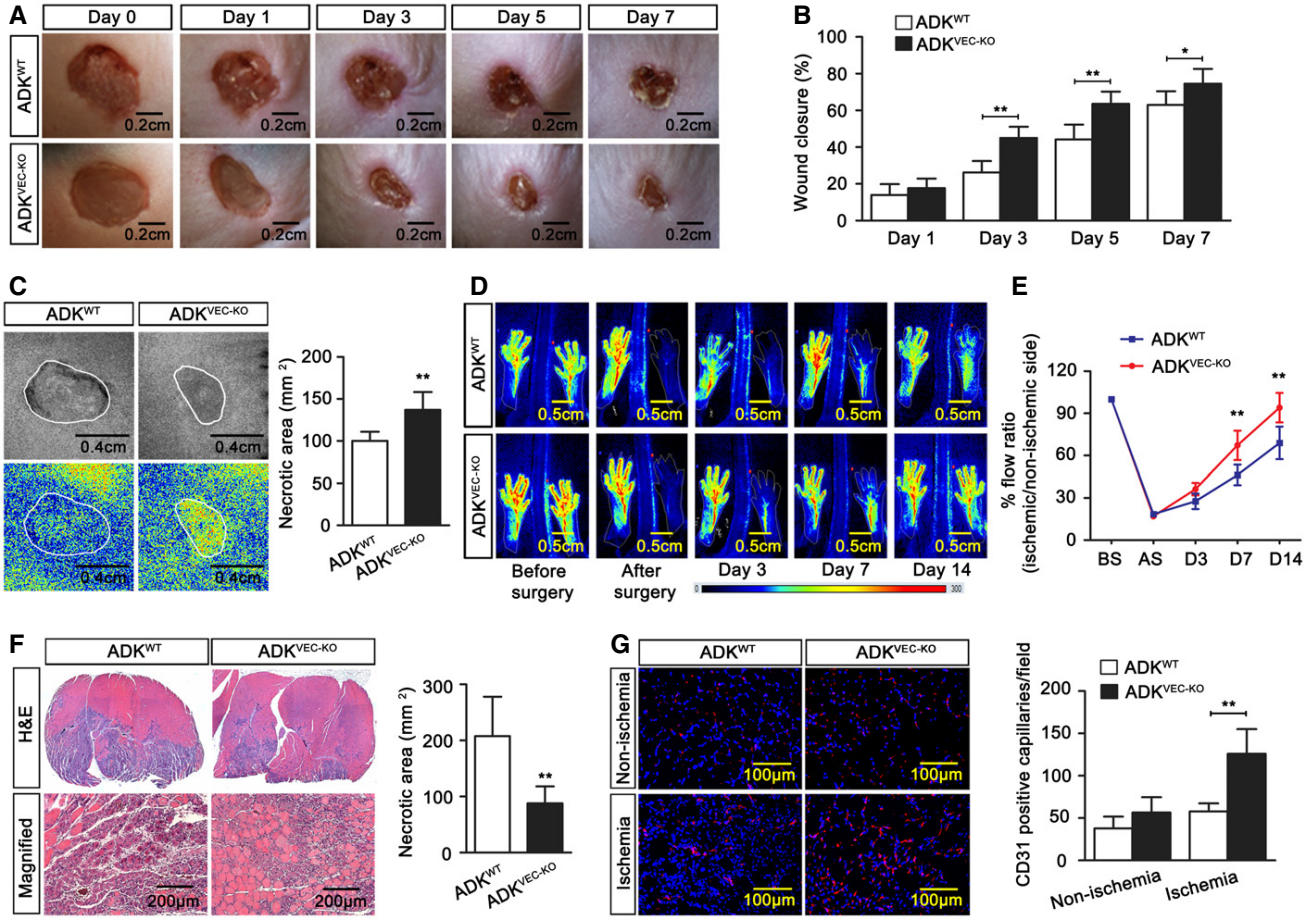
Models of skin wound healing and hindlimb ischemia in mice with endothelial ADK deficiency Representative images of mouse wound healing at different days after skin punch biopsy.Quantification of murine wound area at different time points after injury (*n* = 7 mice per group).Laser speckle contrast imaging and quantification of blood perfusion in murine wounds at day 3 after wounding (*n* = 7 mice per group).Representative laser Doppler perfusion images of mice following unilateral hindlimb ischemia at various days after surgery.Time course of blood perfusion rate examined with computer‐assisted analysis of laser Doppler program (*n* = 7 mice per group).Histological examination of necrotic area in gastrocnemius (GC) muscle at day 7 after ischemia (*n* = 7 mice per group).Representative images and quantification of immunofluorescence staining of CD31 on sections of GC muscle at day 7 after ischemia (*n* = 7 mice per group).Data information: For all bar graphs, data are the means ± SD, *P <* *0.05, and **P <* *0.01 for ADK^VEC‐KD^ vs. ADK^WT^; two‐way ANOVA with Bonferroni's *post‐hoc* test for (B and E); unpaired, two‐tailed Student's *t*‐test for (C, F and G). The exact *P*‐values are specified in Appendix Table S1.

Angiogenesis is critical, although not sufficient, for wound repair due to its role in suppling oxygen and nutrients which support the growth and function of reparative cells in damaged tissues (Eming *et al*, 2007; Lanahan *et al*, 2014). We next evaluated the role of ADK deficiency in the wound healing response in skin. In agreement with the *in vitro* results showing increased migration and proliferation of ADK KD endothelial cells, the wound closed much faster in ADK^VEC‐KO^ mice than littermate controls over a period of 7 days (Fig 5A and B). More importantly, the blood flow in the healing wounds, measured with a laser Doppler, was 28% higher in ADK^VEC‐KO^ mice than in littermate controls (Fig 5C).

Individuals with cardiovascular or metabolic diseases have a blunted angiogenic response to ischemia that is accompanied with poor clinical outcomes (Arenillas *et al*, 2007; Hazarika *et al*, 2007). We next further evaluated the impact of ADK deficiency on perfusion recovery after ischemic injury *in vivo* by introducing the mouse hindlimb ischemic model in which blood flow recovered to 68% of the pre‐surgical flow in littermate controls within 2 weeks following ligation and excision of mouse femoral arteries. However, in ADK^VEC‐KO^ mice, the blood flow recovered almost completely (Fig 5D and E). Histology showed that, in addition to the decreased injured and necrotic area in ischemic gastrocnemius muscles from ADK^VEC‐KO^ mice (Fig 5F), the density of CD31‐expressing endothelial cells was also markedly increased (Fig 5G). Also, endothelium‐dependent dilation to acetylcholine (Ach) was significantly increased (Appendix Fig S3A), whereas constriction to serotonin (5‐HT) was reduced in arterioles of ADK^VEC‐KO^ mice when compared to arteriolar responses in ADK^WT^ mice (Appendix Fig S3B). The increased angiogenesis and arteriolar dilation may not be attributed to a systemic effect of adenosine because the levels of circulating adenosine in the blood of the above mice were the same (Appendix Fig S3C).

### Intracellular adenosine hypomethylates angiogenic genes at the promoter regions

Endothelial cells prominently expressed adenosine receptor 2A (A_2A_R) and 2B (A_2B_R; Appendix Fig S4A). A_2A_R and A_2B_R have been well known for their pro‐angiogenic function. However, the combination of the A_2A_R antagonist ZM 241385 and A_2B_R antagonist MRS1754 at concentrations that effectively lowered the intracellular cAMP level (Appendix Fig S4B) did not significantly compromise the ADK KD‐induced increment of endothelial sprouting (Appendix Fig S4C) and migration (Appendix Fig S4D). This indicates that other important mechanisms can mediate the pro‐angiogenic effect of ADK KD.

**Figure 6:**
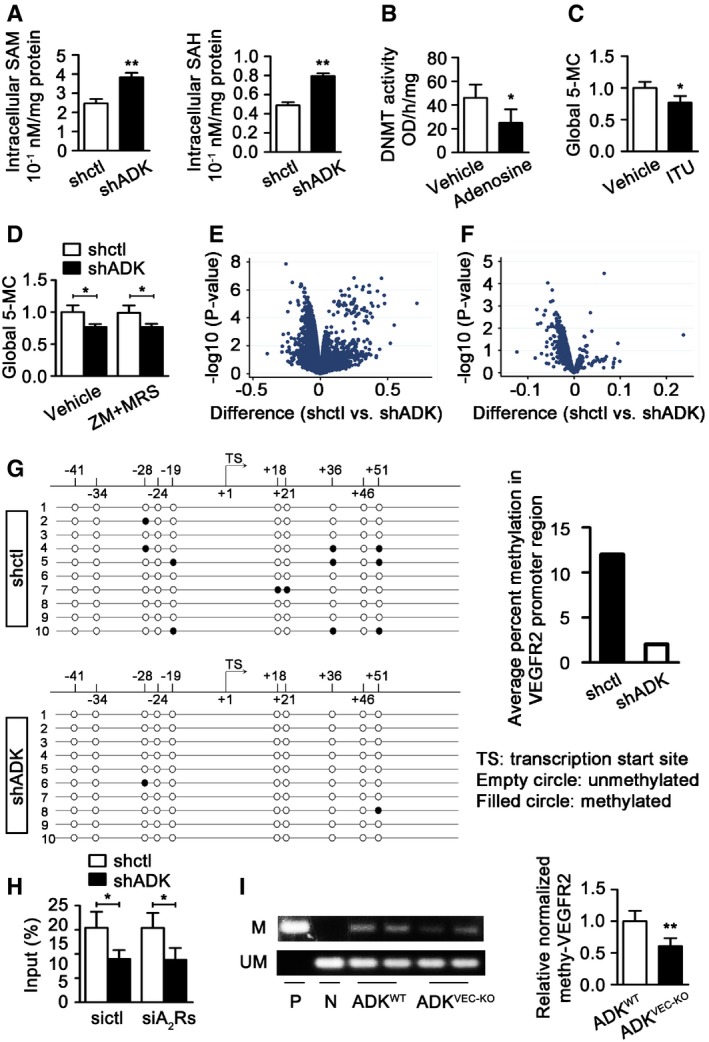
Promoter hypomethylation for angiogenic genes induced by intracellular adenosine Quantification of intracellular SAM and SAH by HPLC in HUVECs. Results are from four independent experiments.Quantification of relative DNMT activity in cell lysates of HUVECs incubated with or without 10 μM adenosine for 24 h. Results are from four independent experiments.Quantification of 5‐mC levels in HUVECs treated with vehicle and ITU (10 μM) for 24 h. Results are from four independent experiments.Quantification of 5‐mC in control and ADK KD HUVECs treated with or without both ZM 241385 and MRS 1754 at 5 μM for 24 h. Results are from four independent experiments.Volcano plot showing standardized mean methylation difference of CpG sites among promoter regions comparing groups of shctl and shADK HUVECs (*n* = 3 independent cultures). *x‐*axis: mean methylation difference of CpG sites between groups of shctl and shADK HUVECs; *y*‐axis: −log_10_ of the *P*‐values.Mean methylation difference of 1,261 CpG sites in the promoters of 109 angiogenic genes comparing groups of shctl and shADK endothelial cells. Number of replicates and axes as in panel (E).(Left) DNA bisulfite analysis results for the region within 100 bp of the transcription start site of VEGFR2. (Right) Average methylation rate of CpG elements of VEGFR2 promoter region revealed by analysis of 10 randomly selected clones.Methylated DNA immunoprecipitation results for VEGFR2 in ADK KD or Ctrl HUVECs transiently transfected with control or A_2A_R/A_2B_R siRNAs. Quantitative PCR was performed with purified total input DNA (input), and the DNA immunoprecipitated with 5‐mC antibody. Results are from four independent experiments.Methylation‐specific PCR analysis of methylation status of VEGFR2 promoter of MAECs. P, positive control; N, negative control. Results are from four independent experiments.Data information: For all bar graphs, data are the means ± SD, *P <* *0.05, and **P <* *0.01; unpaired, two‐tailed Student's *t*‐test for (A–C) and (I); one‐way ANOVA with Tukey's *post‐hoc* test for (D and H). The exact *P*‐values are specified in Appendix Table S1. Source data are available online for this figure.

Since elevated intracellular adenosine shifts the equilibrium of the SAHH‐mediated reaction toward accumulation of SAH, which is a powerful inhibitor of SAM‐dependent transmethylation reactions (Boison, 2013), we hypothesized that the intracellular adenosine induces hypomethylation of angiogenic genes at the promoter regions, thereby boosting their expression and promoting angiogenesis. To test the effect of ADK KD‐induced adenosine elevation on transmethylation reactions, intracellular SAM and SAH were measured using HPLC. ADK KD markedly enhanced the levels of intracellular SAM and SAH (Fig 6A), indicating a decrease in SAM‐dependent transmethylation reactions in ADK KD cells. The influence of adenosine on total DNA methyltransferase (DNMT) activity was directly measured. Adenosine was repeatedly added to the cell medium every 6 h, at a concentration of 20 μM, for 24 h. Nuclear lysates of adenosine‐treated HUVECs, which contained all components of the transmethylation pathway, were collected for measurement of DNMT activity. Treatment with adenosine caused a significant decrease of DNMT activity (Fig 6B), suggesting that indeed adenosine elevation reduces DNMT activity in endothelial cells. In line with these results, the levels of 5‐methylcytosine (5‐mC), an indicator of global DNA methylation, were significantly lower in HUVECs in which ADK was knocked down genetically or inhibited pharmacologically by 5‐iodotubercidin (ITU) at a concentration that effectively elevated the level of intracellular adenosine (Appendix Fig S5A, and Fig 6C and D). Treatment with antagonists of adenosine receptors A_2A_R and A_2B_R did not affect the levels of 5‐mC in ADK KD cells (Fig 6D), indicating that activation of adenosine receptors is not necessarily required for the ADK KD‐induced hypomethylation.

To further validate the findings that intracellular adenosine interferes with global DNA methylation, we performed the Infinium methylation assay to determine the genomewide methylation level. Since promoter hypermethylation correlates with gene silencing, we restricted the analysis to probes within promoter regions. Within the promoter region, we found that methylation was lower in the shADK group (Fig 6E), consistent with the prediction from the 5‐mC assay (Fig 6C). We further confirmed the analysis of methylation differences to a group of 109 genes related to pro‐angiogenesis as defined in the program of Gene Set Enrichment Analysis (GSEA). As shown in the volcano plot in Fig 6F and Appendix Table S2, the CpG sites at the promoter regions maintained a dominant methylation decrease in the shADK group compared with the shctl group. Based on statistical analysis of false discovery rates (FDRs) < 0.2, the methylation levels of 18 CpG sites that mapped to 14 angiogenic genes in the shADK group were considered to be significantly decreased (highlighted in green in Appendix Table S2). Vascular endothelial growth factor receptor 2 (VEGFR2) is one of these 14 angiogenic genes, and its methylation levels on 10 CpG sites in the promoter region were all decreased in the shADK group. Using the same approach, we further analyzed the promoter methylation status of 69 anti‐angiogenetic genes as defined by GSEA. Although the CpG sites at the promoter regions still maintained a relative decrease in methylation in the shADK group (Appendix Table S3), only one CpG site, which mapped to the GDF2 gene, showed significantly decreased methylation (highlighted in green in Appendix Table S3). These data indicate that an elevated intracellular adenosine level by ADK KD preferentially hypomethylates pro‐angiogenic genes in their promoter regions.

To validate the general robustness of the Infinium methylation assay dataset, bisulfite sequencing of VEGFR2 was performed. Within 100 bp of the transcription start site, the average methylation rate of CpG elements in ADK KD endothelial cells was reduced by 90% compared with that in control cells (Fig 6G). To further quantitatively assess the promoter methylation for VEGFR2, methylated DNA was immunoprecipitated by 5‐mC antibody from ADK KD and control HUVECs, and the levels of promoter methylation were quantified by RT–PCR. The level of methylated VEGFR2 was much lower in the shADK group than in the control group, and this reduction was preserved with deficiency of A_2A_R and A_2B_R (Appendix Fig S5B and Fig 6H). Furthermore, in a methylation‐specific PCR assay, the relative level of methylated VEGFR2 was significantly decreased in MAECs from ADK^VEC‐KO^ mice compared to those from control mice (Fig 6I). Thus, these findings show that increasing the intracellular adenosine level via suppressing ADK expression can affect DNA methylation status in endothelial cells by direct biochemical interference with the transmethylation pathway.

### Intracellular adenosine upregulates VEGFR2 expression and signaling by DNA hypomethylation

**Figure 7:**
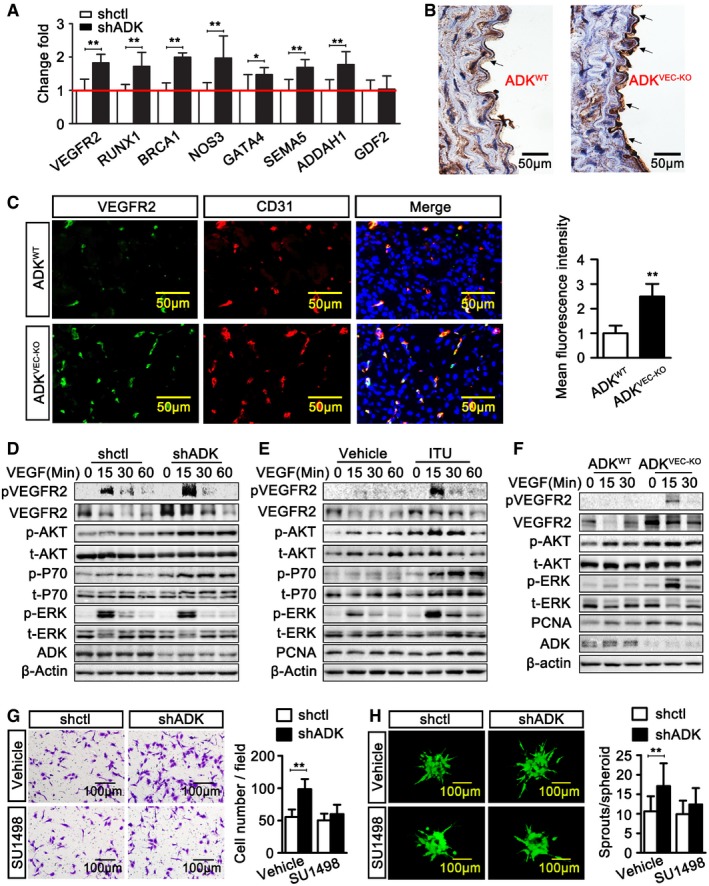
Increased expression of angiogenic genes by intracellular adenosine AReal‐time PCR analysis of angiogenic gene expression in control and ADK KD HUVECs. Results are from four independent experiments.BRepresentative images of VEGFR2 immunohistochemical staining on aortic sections of ADK^WT^ and ADK^VEC‐KD^ mice (*n* = 3 mice per group). The arrows indicate VEGFR2‐positive areas.CImmunofluorescent staining analysis of CD31 and VEGFR2 on sections of GC muscle at day 7 after hindlimb ischemia (*n* = 7 mice per group).D–FConfluent serum‐starved cells were incubated with VEGF‐A at 50 ng/ml. Western blot analysis of VEGFR2, Akt, P70, ERK, and their phosphorylated forms in: (D) control and ADK KD HUVECs, (E) vehicle and 5‐iodotubercidin (ITU)‐treated HUVECs, or (F) MAECs. Images are representative from three independent experiments.GEndothelial migration in control and ADK KD HUVECs treated with or without SU1498 at 1 μM and cultured in a transwell plate for 16 h. Results are from four independent experiments.HEndothelial sprouting of control and ADK KD HUVECs treated with or without SU1498 at 1 μM in a spheroid assay (*n* = 10 spheroids per group).Data information: For all bar graphs, data are the means ± SD, *P <* *0.05, and **P <* *0.01; unpaired, two‐tailed Student's *t*‐test for (A and C); one‐way ANOVA with Tukey's *post‐hoc* test for (G and H). The exact *P*‐values are specified in Appendix Table S1. Source data are available online for this figure.

To determine whether promoter hypomethylation correlates with upregulation of gene expression, we examined the mRNA levels of the 14 pro‐angiogenic genes and the one anti‐angiogenic gene using RT–PCR. Corresponding with the promoter hypomethylation, increased mRNA levels were found for the pro‐angiogenic genes VEGFR2, GATA4, NOS3, DDAH1, RUNX1, Sema5a, and BRCA1 in the shADK group (Fig 7A). However, the mRNA level of the anti‐angiogenic gene GDF2 was not increased in the shADK group. Immunostaining showed an increase of VEGFR2 in aortic endothelium of ADK^VEC‐KO^ mice when compared with that of control mice (Fig 7B). In the mouse ischemic hindlimb, in addition to an increased number of endothelial cells occurring in the ischemic area, the level of endothelial VEGFR2 was also enhanced significantly in ADK^VEC‐KO^ mice compared with control mice (Fig 7C). Corresponding with the increased VEGFR2 expression in ADK KD endothelial cells, VEGFR2 signaling was also enhanced. After incubation with VEGF, the levels of phosphorylated VEGFR2 (p‐VEGFR2) in ADK KD HUVECs were dramatically elevated compared with those in control cells. Moreover, the levels of VEGFR2 downstream molecules, including phosphorylated Akt and P70 S6 kinase (p70S6K, p70), were also markedly increased in ADK KD HUVECs compared to those in control cells (Fig 7D). The above increased VEGFR2 signaling also occurred in MAECs from ADK^VEC‐KO^ mice and HUVECs treated with the ADK inhibitor ITU (Fig 7E and F). The VEGFR2 inhibitor SU1498 dose dependently suppresses angiogenesis (Ferla *et al*, 2011). SU1498, at a low dose (1 μM), did not significantly affect migration and capillary network formation of control HUVECs but significantly suppressed the increased migration (Fig 7G), sprouting (Fig 7H), and capillary network formation (Appendix Fig S5C) of ADK KD HUVECs, indicating that increased VEGFR2 signaling via hypomethylation is one of the major causes of the increased angiogenesis by intracellular adenosine. Since several other pro‐angiogenic factors are upregulated by ADK KD, it seems counterintuitive for the sufficiency of VEGFR2 inhibitor to significantly suppress the increased tube formation and migration of ADK KD cells. Nevertheless, this might be explained by the fundamental role of VEGFR2 in angiogenesis and the potential cross talk among VEGFR2 and other angiogenic factors.

**Figure 8:**
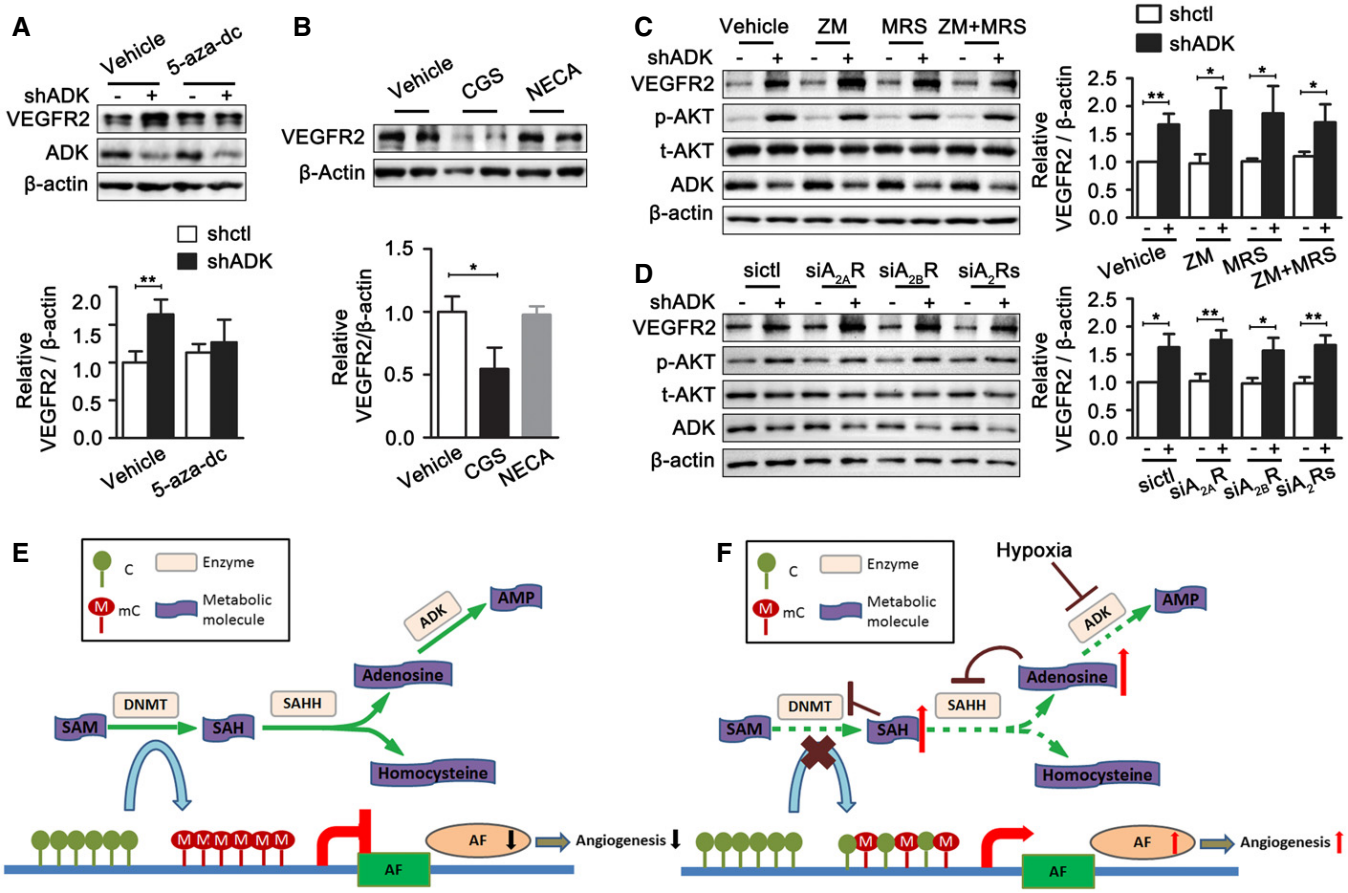
The involvement of DNA hypomethylation or adenosine receptor in VEGFR2 expression AWestern blots of endothelial cell VEGFR2. HUVECs were first infected with adenoviral ADK shRNA or control shRNA and then treated 6 h later with 5‐aza‐dC at 2 μM for 48 h. Results are from three independent experiments.BWestern blots of VEGFR2 expression in HUVECs treated with CGS 21680 at 5 μM or NECA at 5 μM for 24 h. Results are from four independent experiments.CWestern blot analysis of VEGFR2, p‐Akt, and Akt in control and ADK KD HUVECs in the presence of ZM 241385 (5 μM), MRS 1754 (5 μM), or both ZM 241385 and MRS 1754 for 24 h. Results are from four independent experiments.DWestern blot analysis of VEGFR2, p‐Akt, and Akt in control and ADK KD HUVECs transduced with either siA_2A_R or siA_2B_R or both. Results are from four independent experiments.E, FSchematic of the proposed mechanisms for transcriptional regulation of angiogenic factors before (E) and after (F) ADK KD or inhibition. SAM indicates *S*‐adenosylmethionine; DNMT, DNA methyltransferase; SAH, *S*‐adenosylhomocysteine; SAHH, SAH hydrolase; AF, angiogenic factor; C, cytosine; mC, methylated cytosine.Data information: For all bar graphs, data are the mean ± SD, *P <* *0.05, and **P <* *0.01 for indicated comparisons; one‐way ANOVA with Tukey's *post‐hoc* test for (A and B); unpaired, two‐tailed Student's *t*‐test for (C and D). The exact *P*‐values are specified in Appendix Table S1. Source data are available online for this figure.

To further confirm that promoter hypomethylation is a prerequisite for ADK deficiency‐induced upregulation of VEGFR2, HUVECs were first treated with 5‐aza‐2′‐deoxycytidine, a DNMT inhibitor, followed by treatment with adenoviral ADK shRNA. VEGFR2 expression was not increased by ADK KD in HUVECs in which DNA methylation was pre‐suppressed (Fig 8A). To examine whether the activation of adenosine receptors is also necessary for the increase in VEGFR2 upon ADK KD, HUVECs were treated with either NECA, a broad agonist for A_2A_R and A_2B_R, or CGS 21680, a selective A_2A_R agonist, at concentrations that raised the intracellular cAMP level (Appendix Fig S1B). NECA treatment did not affect VEGFR2 expression, and CGS 21680, rather than increasing it, decreased VEGFR2 expression (Fig 8B). In addition, the enhanced expression and signaling of VEGFR2 in ADK KD HUVECs were not compromised by blockade or siRNA KD of A_2A_R, A_2B_R, or both (Fig 8C and D), indicating that the activation of adenosine receptors is not necessarily required for VEGFR2 upregulation induced by ADK deficiency.

## Discussion

In this study, we described a novel epigenetic mechanism by which intracellular adenosine level regulates angiogenesis. Elevated intracellular adenosine level via ADK downregulation in the setting of hypoxia inhibited the transmethylation pathway, decreased methylation of the promoters of pro‐angiogenic genes, and increased expression and signaling of angiogenic factors (Fig 8E and F).

HIF‐1α‐dependent ADK repression and elevation of intracellular adenosine level are among the vascular adaptations to hypoxic stress. Angiogenesis is a major adaptive response to tissue hypoxia. Hypoxia‐induced HIF‐1α accumulation initiates the expression of growth factors and upregulates multiple pro‐angiogenic pathways that mediate key aspects of endothelial biology (Fong, 2008; Krock *et al*, 2011). Furthermore, recent studies show that HIF‐1α‐driven glycolytic reprogramming, as an adaptive metabolic response, controls endothelial proliferation and vessel sprouting (De Bock *et al*, 2013; Xu *et al*, 2014). The findings in our current study demonstrate that hypoxia elevates the intracellular adenosine level through HIF‐1α‐dependent ADK repression. This is consistent with previous work showing that hypoxia, associated with a functional inhibition of ADK, inhibits the intracellular metabolism of adenosine into AMP (Decking *et al*, 1997; Morote‐Garcia *et al*, 2008). In this study, ADK repression‐induced elevation of intracellular adenosine promotes endothelial cell proliferation and migration *in vitro* and vascular growth *in vivo*. From this standpoint, it is likely that elevating intracellular adenosine level through ADK repression in endothelial cells is one of the major mechanisms for HIF‐1α‐dependent vascular adaptation to conditions of limited oxygen availability.

DNA hypomethylation contributes to intracellular adenosine‐induced angiogenesis. ADK KD or inhibition elevates the intracellular adenosine level, causing negative feedback inhibition to transmethylation reactions in endothelial cells, as it does in the brain (Williams‐Karnesky *et al*, 2013). Epigenetic regulation, including DNA methylation, is critically involved in the modulation of endothelial functions (Cooper & Keaney, 2011; Rao *et al*, 2011; Dunn *et al*, 2014, 2015). Several endothelium‐specific genes, such as eNOS, CD31, von Willebrand factor (vWF), VE‐cadherin, and VEGFR2, exhibit different profiles of DNA methylation in their proximal promoter regions (Chan *et al*, 2004; Matouk & Marsden, 2008; Shirodkar *et al*, 2013). The previous study has demonstrated that, in the setting of hypoxia or nutrient deprivation, the VEGFR2‐Akt‐eNOS axis is restricted by DNA methylation via methyl‐CpG‐binding domain protein 2 (MBD2) binding to CpG sites. As a critical angiogenic factor, VEGFR2 gene demethylation by KD or deletion of the MBD2 gene increased VEGFR2 expression and signaling, resulting in accelerated endothelial proliferation and migration *in vitro* as well as fast angiogenesis *in vivo* (Cooper & Keaney, 2011; Rao *et al*, 2011). Our current study further enriches the epigenetic profiles of angiogenesis. ADK is widely accepted to favor SAM‐dependent transmethylation reactions by removing the intracellular adenosine; in this study, we cannot rule out the possibility that ADK can directly affect DNA methylation independently of intracellular adenosine. However, this does not hinder us from concluding that intracellular adenosine promotes angiogenesis via DNA hypomethylation of angiogenic genes, because exogenous adenosine‐induced angiogenesis may also occur partially through this epigenetic pathway.

Exogenous adenosine‐induced angiogenesis may also occur partly through this epigenetic pathway. Several studies have demonstrated that administration of adenosine increases endothelial cell proliferation and migration (Dusseau *et al*, 1986; Ethier *et al*, 1993; Ethier & Dobson, 1997; Sexl *et al*, 1997; Grant *et al*, 1999). However, the mechanisms by which adenosine stimulates angiogenesis have been disputed. For example, Ethier *et al* have found that adenosine‐induced [3H] thymidine uptake by HUVECs is not mimicked by agonists of adenosine receptors or inhibited by antagonists of adenosine receptors (Ethier & Dobson, 1997). Gu *et al* (2000) have used equimolar concentrations of the ADK inhibitor GP‐515 and adenosine to stimulate HUVECs and found that GP‐515, which is less efficacious than adenosine in stimulating VEGF release, causes a greater increase in DNA synthesis and cell proliferation. Thus, Gu *et al* have proposed that the ADK inhibition‐induced high level of intracellular adenosine may stimulate endothelial cell growth. In the present studies, blocking inward transport of adenosine using an ENT inhibitor abrogates exogenous adenosine‐induced VEGFR2 expression (Appendix Fig S6A–C). Therefore, exogenous adenosine at least partially induces angiogenesis via a mechanism similar to that used by intracellular adenosine. After entering into endothelial cells, adenosine suppresses transmethylation reactions (Appendix Fig S6D and E) and induces hypomethylation of promoters of angiogenic genes, causing upregulation of angiogenic factors and therefore promotion of angiogenesis (Appendix Fig S6A, F and G).

VEGFR2 signaling is a vital factor for intracellular adenosine‐mediated angiogenesis. Endothelial VEGFR2 expression and its signaling cascades are required for endothelial cell proliferation and migration, two critical aspects of angiogenesis (Carmeliet, 2000; Potente *et al*, 2011; Pasula *et al*, 2012). We found that VEGFR2 expression and signaling, especially the PI3K/mTOR signaling in endothelial cells with ADK deletion or inhibition, is markedly upregulated. Previous studies have shown that activation of the mTOR signaling pathway contributes to islet β‐cell proliferation and cardiomyocyte hypertrophy induced by ADK inhibition (Fassett *et al*, 2011; Annes *et al*, 2012). Our study dissected the relationship among ADK, intracellular adenosine, and mTOR activation at the epigenetic level.

Deficiency or inhibition of ADK may promote angiogenesis through other mechanisms. The role of adenosine receptors, especially A_2A_R and A_2B_R, in angiogenesis has been well described (Grant *et al*, 1999; Dubey *et al*, 2002; Afzal *et al*, 2003; Montesinos *et al*, 2004). Since the inhibition or deficiency of ADK causes cellular efflux of adenosine, it is possible that the growth‐promoting effect of ADK KD on endothelial cells may also be mediated by autocrine adenosine signaling, especially *in vivo*, although the antagonism of both A_2A_R and A_2B_R did not block the pro‐angiogenic effect of ADK deficiency *in vitro*. In addition, paracrine/autocrine adenosine signaling would be much more anticipated in *in vivo* studies. Under *in vivo* conditions, particularly during hypoxia, adenosine flux is predominantly directed from the extracellular to the intracellular space (Saito *et al*, 1999). The increased intracellular adenosine levels due to ADK inhibition would decrease the transcellular adenosine gradient, thereby decreasing the flux through bidirectional ENTs and, thus, elevating extracellular adenosine levels, which may exert paracrine and autocrine angiogenic functions on most cell types.

The epigenetic pathway revealed in this study may bring a marked change in the use of adenosine or its related medicines in patients. Clinically, the beneficial effects of extracellular or interstitial adenosine on platelet activation and arterial dilation have been emphasized, based on favorable effects of nucleoside transporter inhibitors such as dipyridamole and ticagrelor (Bomberger *et al*, 1982; Cattaneo *et al*, 2014). Our current study indicates that as an adaptive response to hypoxia, elevated intracellular adenosine upon ADK repression is important for endothelial cell proliferation/survival. Since ADK inhibition increases both intracellular and extracellular adenosine levels, targeting ADK could be a very promising strategy to treat ischemic vascular diseases. ADK inhibitors were developed many years ago, but clinical development of these inhibitors was halted due to cerebral hemorrhage (McGaraughty *et al*, 2005). Mice deficient in endothelial ADK do not show such vascular defects. More studies on the effect of ADK on vascular function in other vascular cells in mice are required to demonstrate whether ADK is a promising target in the treatment of vascular diseases.

## Materials and Methods

### Cell culture and treatments

Human umbilical vein endothelial cells (HUVECs) at a passage of 3–8 and mouse aortic endothelial cells (MAECs) at a passage of 2–4 were cultured in endothelial growth medium 2 (EGM‐2). Chemical inhibitors or activators were added (as indicated) to the culture medium of endothelial cells in some experiments. In some experiments, HUVECs were transduced with adenovirus (10 pfu/cell) and were used for experiments 36 h after the transduction. The GFP‐labeled‐ADK shRNA adenovirus targeting the 3′ UTR sequence of human ADK and the control adenovirus were constructed commercially.

### Analysis of ADK, VEGFR2, and other molecules at the mRNA and protein levels

Quantitative real‐time RT–PCR (qRT–PCR) was used to determine mRNA levels. All sequences of primers for the tested molecules are detailed in Appendix Table S4. Western blotting was performed to determine the protein levels of these molecules. Details of qRT–PCR and Western blotting are provided in the Appendix Supplementary Methods. VEGFR2 expression in tissue sections was examined with immunohistochemistry as described in detail in the Appendix Supplementary Methods.

### *In vitro* angiogenesis assays

Assays of *in vitro* tube formation and chemotactic migration with endothelial cells were performed as described previously. Details are provided in the Appendix Supplementary Methods.

### Mouse generation and breeding

The use of experimental animals was approved by the IACUC at the Augusta University in accordance with NIH guidelines. ADK‐floxed (ADK^WT^) control mice were generated by insertion of loxP sites on both sides of ADK exon seven (Fig 3A and B). ADK^WT^ mice were then crossed with Cdh5‐Cre mice, a mouse line in which Cre is selectively expressed in Cdh5 (VE‐cadherin)‐expressing endothelial cells. The resulting ADK^flox/flox^; Cdh5‐Cre (ADK^VEC‐KO^) mice have ADK deficiency in endothelial cells (Fig 3C and D) and were used in the study. Details are provided in the Appendix Supplementary Methods.

### Mouse *in vivo* angiogenesis models

Models of mouse retinal vasculature development, skin wound healing, and hindlimb ischemia were performed using methods previously described (Rao *et al*, 2011; Lanahan *et al*, 2014). Details are provided in the Appendix Supplementary Methods.

### DNMT activity assay and global DNA methylation measurements with MethylFlash methylated DNA 5‐mC quantification kit

DNMT activity of freshly isolated nuclear proteins was quantified using a fluorimetric EpiQuick DNMT Activity Assay Ultra kit per the manufacturer's instruction. In a separate experiment, global DNA methylation status was assessed using the MethylFlash Methylated DNA quantification kit.

### Infinium methylation assay

The bisulfite conversion of genomic DNA was conducted using EZ DNA Methylation Gold kit (Zymo Research). 200 ng converted DNA was analyzed using Illumina Infinium 450K Methylation array according to the manufacturer's suggested protocols (Illumina). Details are provided in the Appendix Supplementary Methods.

### Methylated DNA immunoprecipitation (MeDIP)–qRT–PCR

MeDIP–qRT–PCR was carried out with a Magnetic Methylated DNA Immunoprecipitation kit (Diagenode, Denville, NJ, USA). The MeDIP DNA was used for qPCR. PCR was performed as described in the Diagenode MeDIP manual.

### DNA extraction and methylation‐specific PCR (MSP)

DNA was extracted from MAECs using a DNeasy Blood and Tissue Kit. Methylation status was determined by methylation‐specific PCR using a methylation kit. MethPrimer software was used for prediction of the CpG island of VEGFR2 and design of methylation‐specific primers. ImageJ was used for semiquantitative measurement of methylated and unmethylated VEGFR2. Methylated VEGFR2 was normalized by comparison with unmethylated VEGFR2. Details are provided in the Appendix Supplementary Methods.

### DNA bisulfite sequencing analysis

Genomic DNA was bisulfite converted by an EpiTect Bisulfite Kit (QIAGEN, Venlo, the Netherlands), followed by PCR amplification of targeted sequences. The resulting PCR products were directly cloned into a TA vector. The methylation state of each targeted sequence was then analyzed by DNA sequencing.

### Measurement of intracellular adenosine, SAM, and SAH levels

Adenosine, SAM, and SAH were measured with reversed‐phase high‐performance liquid chromatography. The assay is detailed in the Appendix Supplementary Methods.

### Statistical analysis

Animal numbers and sample sizes which reflected the minimal number needed for statistical significance were determined by power analysis and prior experience. No data were excluded from any of the experiments. For animal studies, grouping was performed based on animal age and genotype with no randomization used. The data are presented as the mean ± SD and were analyzed by one‐way ANOVA followed by Tukey's *post‐hoc* test or Student's *t*‐test to evaluate two‐tailed levels of significance. Two‐way repeated‐measures ANOVA with Bonferroni's *post‐hoc* test was done to assess wound size and perfusion improvement over time within groups. The null hypothesis was rejected at *P *≤* *0.05.

## Author contributions

YX and YH conceived the study, designed all of the biological experiments, and analyzed the data. YX performed the animal experiments with assistance from YW, ZL, XA, LW, and YC. SY performed the *in vitro* experiments with assistance from YX, YW, YZ, XZ, and JX. QY and MNH performed experiments with HPLC assay. YP and XW analyzed methylation assay data. YW, ZB, XJ, QL, MH, and CWeber contributed to study design. YX, DJF, NLW, DB, CWu, and YH wrote the paper. YH supervised all experiments.

## Conflict of interest

The authors declare that they have no conflict of interest.

**The paper explained**

**Problem**

Individuals with peripheral vascular and coronary artery disease present with a blunted angiogenic response to ischemia that is accompanied with poor clinical outcomes. A long‐standing goal in vascular disease research has been to generate therapies that promote angiogenesis in compromised tissue beds. There is evidence that adenosine has a long‐term role in maintaining tissue oxygenation in response to chronic ischemic/hypoxic stress by stimulating angiogenesis. However, the mechanism by which adenosine induces angiogenesis is still poorly understood. In addition, the functional roles of the adenosine‐metabolizing enzyme ADK and intracellular adenosine in angiogenesis have not been investigated.

**Results**

We have demonstrated that hypoxia increases intracellular adenosine through the downregulation of ADK. Targeting ADK to elevate intracellular adenosine promotes endothelial proliferation and migration *in vitro* as well as vessel sprouting *ex vivo*. Additionally, endothelial‐specific ADK knockout mice have increased retinal angiogenesis, accelerated wound healing, and were protected against hindlimb ischemic injury. This study also identified the epigenetic mechanism through which intracellular adenosine and ADK regulate angiogenesis.

**Impact**

This study provides valuable new insights into the epigenetic control of pro‐angiogenic factors by intracellular adenosine and implies that therapeutic augmentation of the adenosine system by targeting ADK is able to stimulate the formation of new blood vessels.

## Supporting information



Appendix

Source Data for Appendix

Review Process File

Source Data for Figure 1

Source Data for Figure 2

Source Data for Figure 3

Source Data for Figure 6

Source Data for Figure 7

Source Data for Figure 8

## References

[emmm201607066-bib-0001] Adair TH (2005) Growth regulation of the vascular system: an emerging role for adenosine. Am J Physiol Regul Integr Comp Physiol 289: R283–R296 16014444 10.1152/ajpregu.00840.2004

[emmm201607066-bib-0002] Afzal A , Shaw LC , Caballero S , Spoerri PE , Lewin AS , Zeng D , Belardinelli L , Grant MB (2003) Reduction in preretinal neovascularization by ribozymes that cleave the A2B adenosine receptor mRNA. Circ Res 93: 500–506 12919950 10.1161/01.RES.0000091260.78959.BC

[emmm201607066-bib-0003] Annes JP , Ryu JH , Lam K , Carolan PJ , Utz K , Hollister‐Lock J , Arvanites AC , Rubin LL , Weir G , Melton DA (2012) Adenosine kinase inhibition selectively promotes rodent and porcine islet beta‐cell replication. Proc Natl Acad Sci USA 109: 3915–3920 22345561 10.1073/pnas.1201149109 PMC3309788

[emmm201607066-bib-0004] Arenillas JF , Sobrino T , Castillo J , Davalos A (2007) The role of angiogenesis in damage and recovery from ischemic stroke. Curr Treat Options Cardiovasc Med 9: 205–212 17601384 10.1007/s11936-007-0014-5

[emmm201607066-bib-0005] Boison D (2013) Adenosine kinase: exploitation for therapeutic gain. Pharmacol Rev 65: 906–943 23592612 10.1124/pr.112.006361 PMC3698936

[emmm201607066-bib-0006] Bomberger RA , DePalma RG , Ambrose TA , Manalo P (1982) Aspirin and dipyridamole inhibit endothelial healing. Arch Surg 117: 1459–1464 7138304 10.1001/archsurg.1982.01380350057008

[emmm201607066-bib-0007] Carmeliet P (2000) Mechanisms of angiogenesis and arteriogenesis. Nat Med 6: 389–395 10742145 10.1038/74651

[emmm201607066-bib-0008] Cattaneo M , Schulz R , Nylander S (2014) Adenosine‐mediated effects of ticagrelor: evidence and potential clinical relevance. J Am Coll Cardiol 63: 2503–2509 24768873 10.1016/j.jacc.2014.03.031

[emmm201607066-bib-0009] Chan Y , Fish JE , D'Abreo C , Lin S , Robb GB , Teichert AM , Karantzoulis‐Fegaras F , Keightley A , Steer BM , Marsden PA (2004) The cell‐specific expression of endothelial nitric‐oxide synthase: a role for DNA methylation. J Biol Chem 279: 35087–35100 15180995 10.1074/jbc.M405063200

[emmm201607066-bib-0010] Cooper MP , Keaney JF Jr (2011) Epigenetic control of angiogenesis via DNA methylation. Circulation 123: 2916–2918 21670233 10.1161/CIRCULATIONAHA.111.033092

[emmm201607066-bib-0011] De Bock K , Georgiadou M , Schoors S , Kuchnio A , Wong BW , Cantelmo AR , Quaegebeur A , Ghesquiere B , Cauwenberghs S , Eelen G *et al* (2013) Role of PFKFB3‐driven glycolysis in vessel sprouting. Cell 154: 651–663 23911327 10.1016/j.cell.2013.06.037

[emmm201607066-bib-0012] Decking UK , Schlieper G , Kroll K , Schrader J (1997) Hypoxia‐induced inhibition of adenosine kinase potentiates cardiac adenosine release. Circ Res 81: 154–164 9242176 10.1161/01.res.81.2.154

[emmm201607066-bib-0013] Drabikowska AK , Halec L , Shugar D (1985) Purification and properties of adenosine kinase from rat liver: separation from deoxyadenosine kinase activity. Z Naturforsch C 40: 34–41 2986372 10.1515/znc-1985-1-209

[emmm201607066-bib-0014] Drury AN , Szent‐Gyorgyi A (1929) The physiological activity of adenine compounds with especial reference to their action upon the mammalian heart. J Physiol 68: 213–237 16994064 10.1113/jphysiol.1929.sp002608 PMC1402863

[emmm201607066-bib-0015] Dubey RK , Gillespie DG , Jackson EK (2002) A(2B) adenosine receptors stimulate growth of porcine and rat arterial endothelial cells. Hypertension 39: 530–535 11882603 10.1161/hy0202.103075

[emmm201607066-bib-0016] Dunn J , Qiu H , Kim S , Jjingo D , Hoffman R , Kim CW , Jang I , Son DJ , Kim D , Pan C *et al* (2014) Flow‐dependent epigenetic DNA methylation regulates endothelial gene expression and atherosclerosis. J Clin Invest 124: 3187–3199 24865430 10.1172/JCI74792 PMC4071393

[emmm201607066-bib-0017] Dunn J , Thabet S , Jo H (2015) Flow‐dependent epigenetic DNA methylation in endothelial gene expression and atherosclerosis. Arterioscler Thromb Vasc Biol 35: 1562–1569 25953647 10.1161/ATVBAHA.115.305042 PMC4754957

[emmm201607066-bib-0018] Dusseau JW , Hutchins PM , Malbasa DS (1986) Stimulation of angiogenesis by adenosine on the chick chorioallantoic membrane. Circ Res 59: 163–170 2427248 10.1161/01.res.59.2.163

[emmm201607066-bib-0019] Eming SA , Brachvogel B , Odorisio T , Koch M (2007) Regulation of angiogenesis: wound healing as a model. Prog Histochem Cytochem 42: 115–170 17980716 10.1016/j.proghi.2007.06.001

[emmm201607066-bib-0020] Ethier MF , Chander V , Dobson JG Jr (1993) Adenosine stimulates proliferation of human endothelial cells in culture. Am J Physiol 265: H131–H138 8342624 10.1152/ajpheart.1993.265.1.H131

[emmm201607066-bib-0021] Ethier MF , Dobson JG Jr (1997) Adenosine stimulation of DNA synthesis in human endothelial cells. Am J Physiol 272: H1470–H1479 9087626 10.1152/ajpheart.1997.272.3.H1470

[emmm201607066-bib-0022] Fantin A , Vieira JM , Plein A , Maden CH , Ruhrberg C (2013) The embryonic mouse hindbrain as a qualitative and quantitative model for studying the molecular and cellular mechanisms of angiogenesis. Nat Protoc 8: 418–429 23424750 10.1038/nprot.2013.015 PMC3763679

[emmm201607066-bib-0023] Fassett JT , Hu X , Xu X , Lu Z , Zhang P , Chen Y , Bache RJ (2011) Adenosine kinase regulation of cardiomyocyte hypertrophy. Am J Physiol Heart Circ Physiol 300: H1722–H1732 21335462 10.1152/ajpheart.00684.2010 PMC3094084

[emmm201607066-bib-0024] Ferla R , Bonomi M , Otvos L Jr , Surmacz E (2011) Glioblastoma‐derived leptin induces tube formation and growth of endothelial cells: comparison with VEGF effects. BMC Cancer 11: 303 21771332 10.1186/1471-2407-11-303 PMC3146945

[emmm201607066-bib-0025] Fong GH (2008) Mechanisms of adaptive angiogenesis to tissue hypoxia. Angiogenesis 11: 121–140 18327686 10.1007/s10456-008-9107-3

[emmm201607066-bib-0026] Fredholm BB (2007) Adenosine, an endogenous distress signal, modulates tissue damage and repair. Cell Death Differ 14: 1315–1323 17396131 10.1038/sj.cdd.4402132

[emmm201607066-bib-0027] Grant MB , Tarnuzzer RW , Caballero S , Ozeck MJ , Davis MI , Spoerri PE , Feoktistov I , Biaggioni I , Shryock JC , Belardinelli L (1999) Adenosine receptor activation induces vascular endothelial growth factor in human retinal endothelial cells. Circ Res 85: 699–706 10521243 10.1161/01.res.85.8.699

[emmm201607066-bib-0028] Gu JW , Ito BR , Sartin A , Frascogna N , Moore M , Adair TH (2000) Inhibition of adenosine kinase induces expression of VEGF mRNA and protein in myocardial myoblasts. Am J Physiol Heart Circ Physiol 279: H2116–H2123 11045944 10.1152/ajpheart.2000.279.5.H2116

[emmm201607066-bib-0029] Hazarika S , Dokun AO , Li Y , Popel AS , Kontos CD , Annex BH (2007) Impaired angiogenesis after hindlimb ischemia in type 2 diabetes mellitus: differential regulation of vascular endothelial growth factor receptor 1 and soluble vascular endothelial growth factor receptor 1. Circ Res 101: 948–956 17823371 10.1161/CIRCRESAHA.107.160630

[emmm201607066-bib-0030] Krock BL , Skuli N , Simon MC (2011) Hypoxia‐induced angiogenesis: good and evil. Genes Cancer 2: 1117–1133 22866203 10.1177/1947601911423654 PMC3411127

[emmm201607066-bib-0031] Lanahan AA , Lech D , Dubrac A , Zhang J , Zhuang ZW , Eichmann A , Simons M (2014) PTP1b is a physiologic regulator of vascular endothelial growth factor signaling in endothelial cells. Circulation 130: 902–909 24982127 10.1161/CIRCULATIONAHA.114.009683 PMC6060619

[emmm201607066-bib-0032] Liem LK , Choong LH , Woo KT (2001) Action of dipyridamole and warfarin on growth of human endothelial cells cultured in serum‐free media. Clin Biochem 34: 141–147 11311224 10.1016/s0009-9120(01)00194-1

[emmm201607066-bib-0033] Loffler M , Morote‐Garcia JC , Eltzschig SA , Coe IR , Eltzschig HK (2007) Physiological roles of vascular nucleoside transporters. Arterioscler Thromb Vasc Biol 27: 1004–1013 17332491 10.1161/ATVBAHA.106.126714

[emmm201607066-bib-0034] Matouk CC , Marsden PA (2008) Epigenetic regulation of vascular endothelial gene expression. Circ Res 102: 873–887 18436802 10.1161/CIRCRESAHA.107.171025

[emmm201607066-bib-0035] McGaraughty S , Cowart M , Jarvis MF , Berman RF (2005) Anticonvulsant and antinociceptive actions of novel adenosine kinase inhibitors. Curr Top Med Chem 5: 43–58 15638777 10.2174/1568026053386845

[emmm201607066-bib-0036] Montesinos MC , Shaw JP , Yee H , Shamamian P , Cronstein BN (2004) Adenosine A(2A) receptor activation promotes wound neovascularization by stimulating angiogenesis and vasculogenesis. Am J Pathol 164: 1887–1892 15161625 10.1016/S0002-9440(10)63749-2 PMC1615751

[emmm201607066-bib-0037] Morin KT , Carlson PD , Tranquillo RT (2015) Automated image analysis programs for the quantification of microvascular network characteristics. Methods 84: 76–83 25843608 10.1016/j.ymeth.2015.03.014 PMC4526423

[emmm201607066-bib-0038] Morote‐Garcia JC , Rosenberger P , Kuhlicke J , Eltzschig HK (2008) HIF‐1‐dependent repression of adenosine kinase attenuates hypoxia‐induced vascular leak. Blood 111: 5571–5580 18309031 10.1182/blood-2007-11-126763

[emmm201607066-bib-0039] Nakatsu MN , Hughes CC (2008) An optimized three‐dimensional *in vitro* model for the analysis of angiogenesis. Methods Enzymol 443: 65–82 18772011 10.1016/S0076-6879(08)02004-1

[emmm201607066-bib-0040] Pasula S , Cai X , Dong Y , Messa M , McManus J , Chang B , Liu X , Zhu H , Mansat RS , Yoon SJ *et al* (2012) Endothelial epsin deficiency decreases tumor growth by enhancing VEGF signaling. J Clin Invest 122: 4424–4438 23187125 10.1172/JCI64537 PMC3533553

[emmm201607066-bib-0041] Pattillo CB , Bir SC , Branch BG , Greber E , Shen X , Pardue S , Patel RP , Kevil CG (2011) Dipyridamole reverses peripheral ischemia and induces angiogenesis in the Db/Db diabetic mouse hind‐limb model by decreasing oxidative stress. Free Radic Biol Med 50: 262–269 21070849 10.1016/j.freeradbiomed.2010.10.714 PMC4413947

[emmm201607066-bib-0042] Potente M , Gerhardt H , Carmeliet P (2011) Basic and therapeutic aspects of angiogenesis. Cell 146: 873–887 21925313 10.1016/j.cell.2011.08.039

[emmm201607066-bib-0043] Rao X , Zhong J , Zhang S , Zhang Y , Yu Q , Yang P , Wang MH , Fulton DJ , Shi H , Dong Z *et al* (2011) Loss of methyl‐CpG‐binding domain protein 2 enhances endothelial angiogenesis and protects mice against hind‐limb ischemic injury. Circulation 123: 2964–2974 21670230 10.1161/CIRCULATIONAHA.110.966408 PMC4120778

[emmm201607066-bib-0044] Saito H , Nishimura M , Shinano H , Makita H , Tsujino I , Shibuya E , Sato F , Miyamoto K , Kawakami Y (1999) Plasma concentration of adenosine during normoxia and moderate hypoxia in humans. Am J Respir Crit Care Med 159: 1014–1018 10051286 10.1164/ajrccm.159.3.9803100

[emmm201607066-bib-0045] Sexl V , Mancusi G , Holler C , Gloria‐Maercker E , Schutz W , Freissmuth M (1997) Stimulation of the mitogen‐activated protein kinase via the A2A‐adenosine receptor in primary human endothelial cells. J Biol Chem 272: 5792–5799 9038193 10.1074/jbc.272.9.5792

[emmm201607066-bib-0046] Shirodkar AV , St Bernard R , Gavryushova A , Kop A , Knight BJ , Yan MS , Man HS , Sud M , Hebbel RP , Oettgen P *et al* (2013) A mechanistic role for DNA methylation in endothelial cell (EC)‐enriched gene expression: relationship with DNA replication timing. Blood 121: 3531–3540 23449636 10.1182/blood-2013-01-479170 PMC3637020

[emmm201607066-bib-0047] Simons M , Alitalo K , Annex BH , Augustin HG , Beam C , Berk BC , Byzova T , Carmeliet P , Chilian W , Cooke JP *et al* (2015) State‐of‐the‐art methods for evaluation of angiogenesis and tissue vascularization: a Scientific Statement From the American Heart Association. Circ Res 116: e99–e132 25931450 10.1161/RES.0000000000000054 PMC8077227

[emmm201607066-bib-0048] Williams‐Karnesky RL , Sandau US , Lusardi TA , Lytle NK , Farrell JM , Pritchard EM , Kaplan DL , Boison D (2013) Epigenetic changes induced by adenosine augmentation therapy prevent epileptogenesis. J Clin Invest 123: 3552–3563 23863710 10.1172/JCI65636 PMC3726154

[emmm201607066-bib-0049] Xu Y , An X , Guo X , Habtetsion TG , Wang Y , Xu X , Kandala S , Li Q , Li H , Zhang C *et al* (2014) Endothelial PFKFB3 plays a critical role in angiogenesis. Arterioscler Thromb Vasc Biol 34: 1231–1239 24700124 10.1161/ATVBAHA.113.303041 PMC4120754

