# Supplementary material for: Intracellular adenosine regulates epigenetic programming in endothelial cells to promote angiogenesis
Source: EMBO Mol Med. 2017 Jul 27;9(9):1263–78. doi: 10.15252/emmm.201607066 (PMC5582416; doi:10.15252/emmm.201607066)
Supplement: Supplementary file 1 — Appendix [file EMMM-9-1263-s001.pdf]

APPENDIX

Intracellular adenosine alters epigenetic programming in endothelial cells to promote angiogenesis

This appendix includes 6 figures, 4 tables and Supplementary methods.

Table of Contents **Page**

Appendix Figure S1. The role of HIFs in hypoxia-induced ADK downregulation. 2

Appendix Figure S2. The effect of ADK KD on endothelial angiogenesis. 4

Appendix Figure S3. Arteriole relaxation and constriction as well as circulating adenosine in ADK^{WT} and $ADK^{VEC\text{-}KO}$ mice. 5

Appendix Figure S4. The role of adenosine receptors in the angiogenic effects of ADK KD. 6

Appendix Figure S5. ADK inhibitor efficiency, adenosine receptor KD efficiency and the effect of VEGFR2 on ADK KD-increased endothelial tube formation. 8

Appendix Figure S6. The effect of exogenous adenosine on DNA methylation, VEGFR2 expression and endothelial functions. 10

Appendix Table S1. Statistical analysis information. 12

Appendix Table S2. Pro-angiogenic genes with increased and decreased methylation upon ADK knockdown. 15

Appendix Table S3. Anti-angiogenic genes with increased and decreased methylation upon ADK knockdown. 20

Appendix Table S4. Primer sequences for qRT-PCR analysis. 23

Appendix Supplementary Methods 25

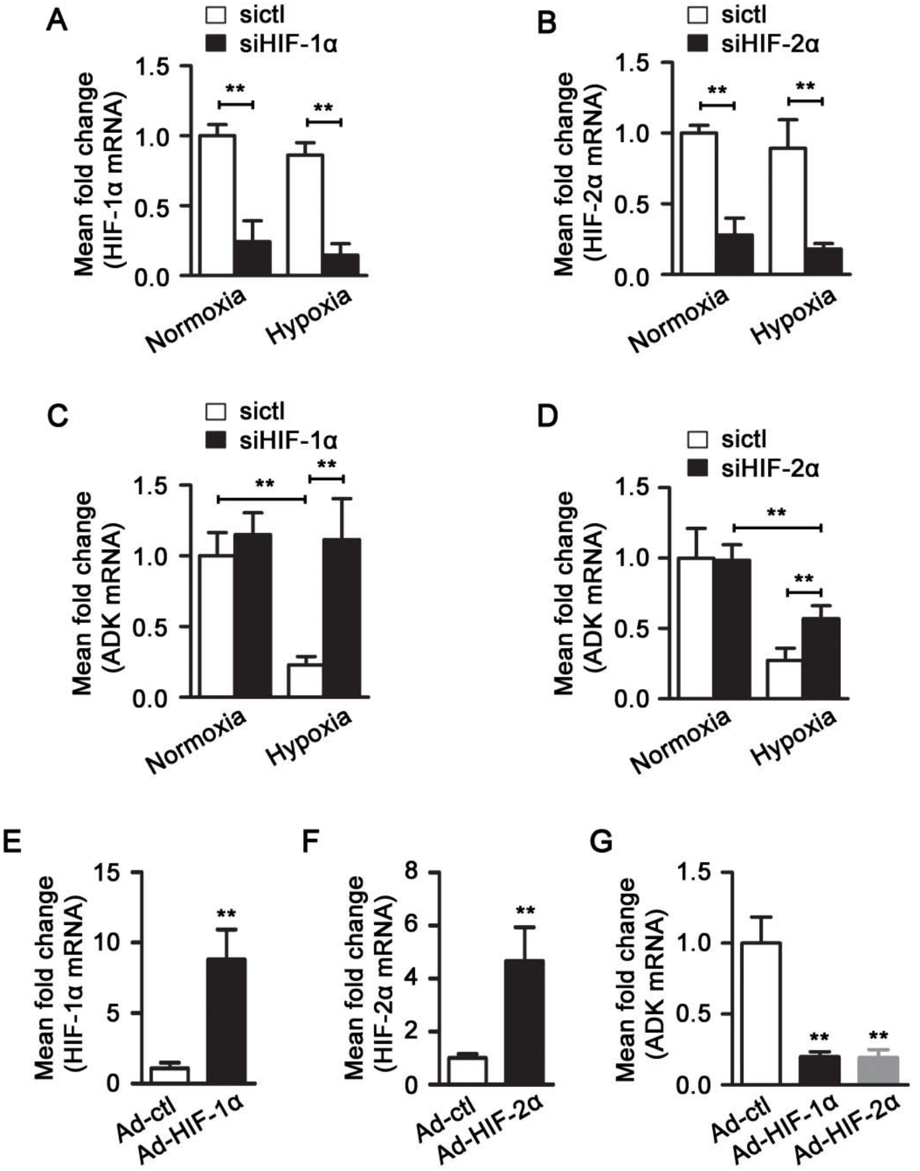

Appendix Figure S1. Hypoxia-induced factors (HIFs) mediates hypoxia-induced ADK downregulation.

A, Real-Time PCR analysis of mRNA level of HIF-1α in HUVECs (n=4). Cells were transiently transfected with siHIF-1α or sictl. Forty-eight hours after transfection, cells were exposed to hypoxia (0.5 % O2) or air (21 % O2) for an additional 24 h.

B, Real-Time PCR analysis of mRNA level of HIF-2α in HUVECs (n=4). Cells were transiently transfected with siHIF-2α or sictl. Forty-eight hours after transfection, cells were exposed to hypoxia (0.5 % O2) or air (21 % O2) for an additional 24 h.

C, Real-Time PCR analysis of mRNA level of ADK in HUVECs (n=4). Cells were transiently transfected with siHIF-1α or sictl. Forty-eight hours after transfection, cells were exposed to hypoxia (0.5 % O2) or air (21 % O2) for an additional 24 h.

D, Real-Time PCR analysis of mRNA level of ADK in HUVECs (n=4). Cells were transiently transfected with siHIF-2α or sictl. Forty-eight hours after transfection, cells were exposed to hypoxia (0.5 % O2) or air (21 % O2) for an additional 24 h.

E, Real-Time PCR analysis of mRNA level of HIF-1α in HUVECs infected with Ad-mutHIF-1α or Ad-Ctrl (n=4).

F, Real-Time PCR analysis of mRNA level of HIF-2α in HUVECs infected with Ad-mutHIF-2α or Ad-Ctrl (n=4).

G, Real-Time PCR analysis of mRNA levels of HIF-1α and HIF-2α in HUVECs infected with Ad-mutHIF-1α, Ad-mutHIF-2α, or Ad-Ctrl (n=4).

Data are the Mean ± SD, **P* <0.05 and ** *P* <0.01 for indicated comparisons (One way ANOVA with Tukey's *post-hoc* test for A-D and G; unpaired, two-tailed Student's *t*-test for E and F). The exact *P*-values are specified in Appendix Table S1.

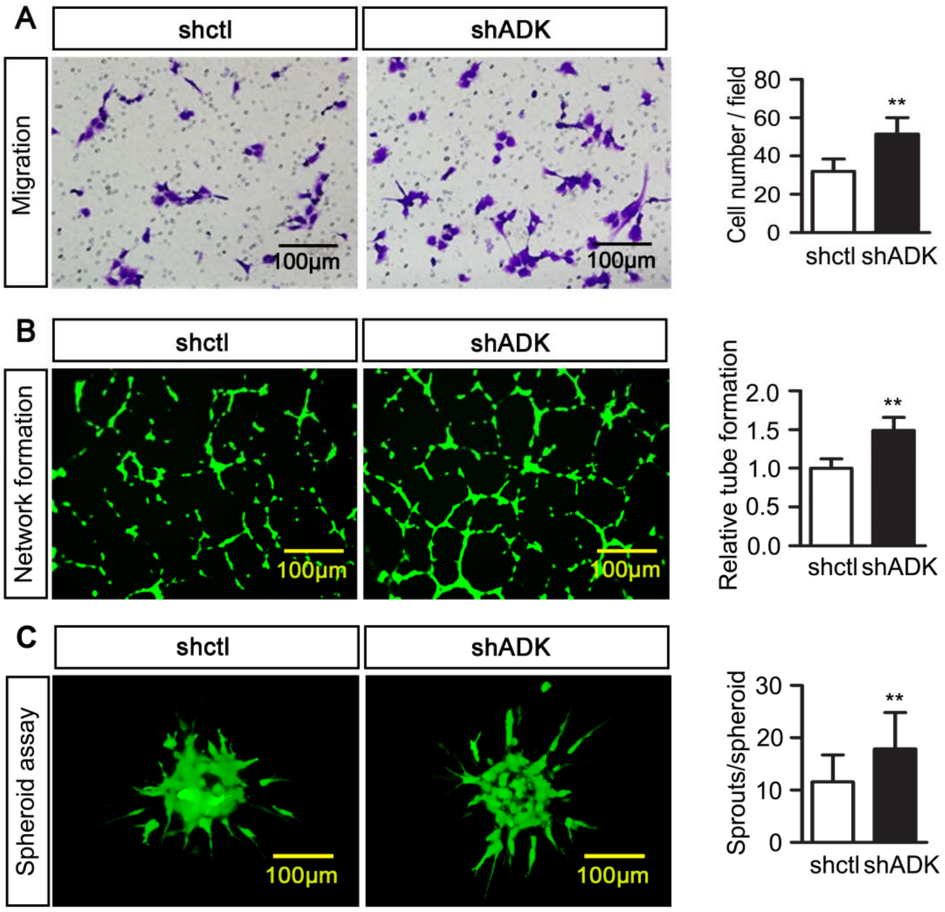

Appendix Figure S2. The effect of ADK KD on endothelial angiogenesis.

A, Representative images and quantification of HUVEC migration to cell medium containing VEGF-A at 50 ng/mL in a transwell assay (n = 3).

B, Representative images and quantification of capillary network formation of HUVECs cultured in growth factor–deprived Matrigel (n = 3).

C, Representative images of spheroid sprouting assay and quantification of sprout numbers for control and ADK KD HUVECs (n = 30 spheroids per group).

All images are representative. For all bar graphs, data are the Mean ± SD, **P* <0.05 and ** *P* <0.01 (Unpaired, two-tailed Student's *t*-test). The exact *P*-values are specified in Appendix Table S1.

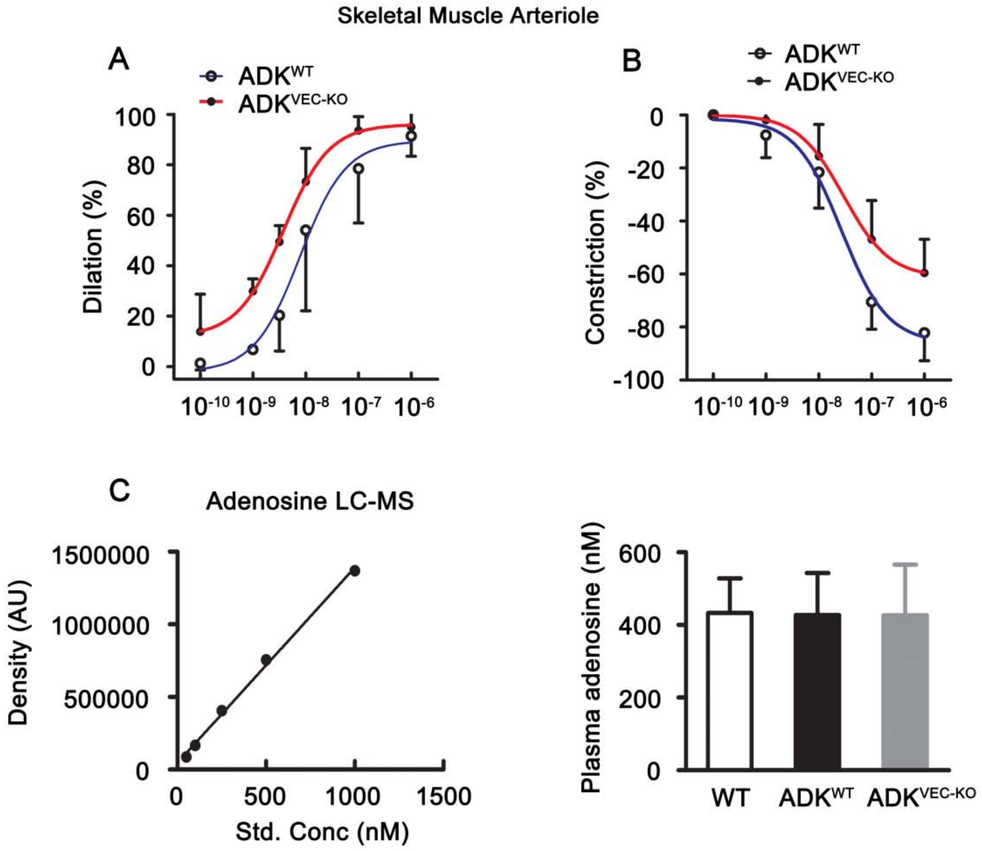

Appendix Figure S3. Arteriole relaxation and constriction as well as circulating adenosine in ADK^{WT} and ADK^{VEC-KO} mice.

A-B, Diameter changes of isolated and pressurized gracilis muscle arterioles of ADK^{WT} and ADK^{VEC-KO} mice in response to the vasodilator ACh (10^{-10} -10^{-6} M, *n* = 5 mice for each group) (**A**), or to the vasoconstrictor 5-HT (10^{-10} -10^{-6} M, *n* = 5 mice for each group) (**B**).

C, Circulating adenosine in ADK^{WT} and ADK^{VEC-KO} mice. Blood was drawn from mice (n=3 mice per group) and the levels of adenosine in plasma analyzed with HPLC.

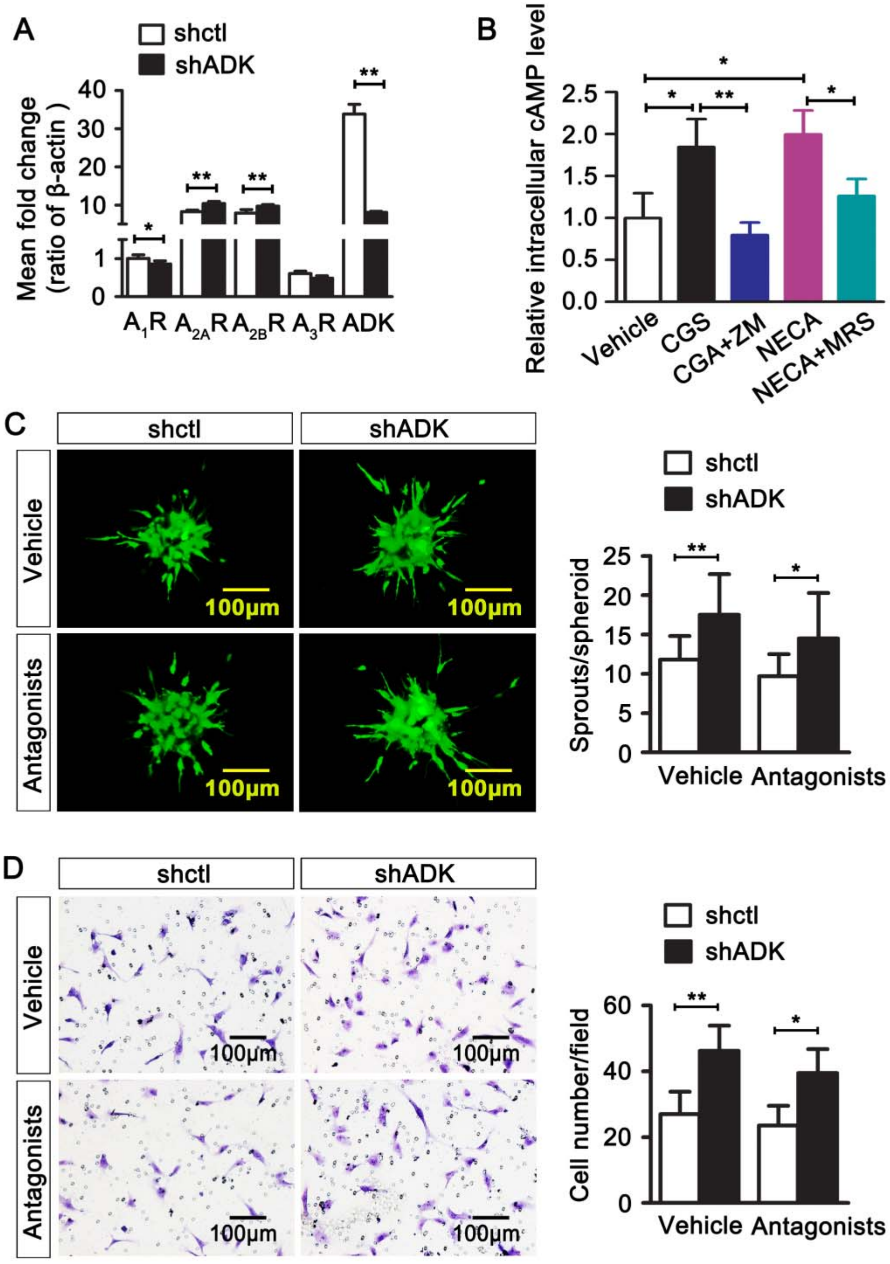

Appendix Figure S4. The role of adenosine receptors in the angiogenic effects of ADK KD.

A, The levels of endothelial A_1R, $A_{2A}R$, $A_{2B}R$ and A_3R were quantified with real time RT-PCR after HUVECs were infected with adenoviral shctl and shADK for 36 hours . Results are from three independent experiments.

B, A commercial kit (Cell Signaling Technology) was used to measure cAMP levels of endothelial cell lysates. HUVECs were pretreated with vehicle or 5 μM ZM 241385 or 5 μM MRS 1754 for 15 min, then followed by 5 μM CGS 21680 and/or 5 μM NECA for 1 hour. Results are from three independent experiments.

C, Representative images and quantification of endothelial sprouting of control and ADK KD HUVECs in a spheroid assay in the absence or presence of both ZM 241385 and MRS 1754 at 5 μM (n=10 spheroids per group).

D, Representative images and quantification of endothelial migration for control and ADK KD HUVECs in a transwell assay in the absence or presence of both ZM 241385 and MRS 1754 at 5 μM. Results are from four independent experiments.

Data information: For all bar graphs, data are the Mean ± SD, * $P < 0.05$ and ** $P < 0.01$ for indicated comparisons (Unpaired, two-tailed Student's *t*-test for A; One-way ANOVA with Tukey's *post-hoc* test for B-D). The exact *P*-values are specified in Appendix Table S1.

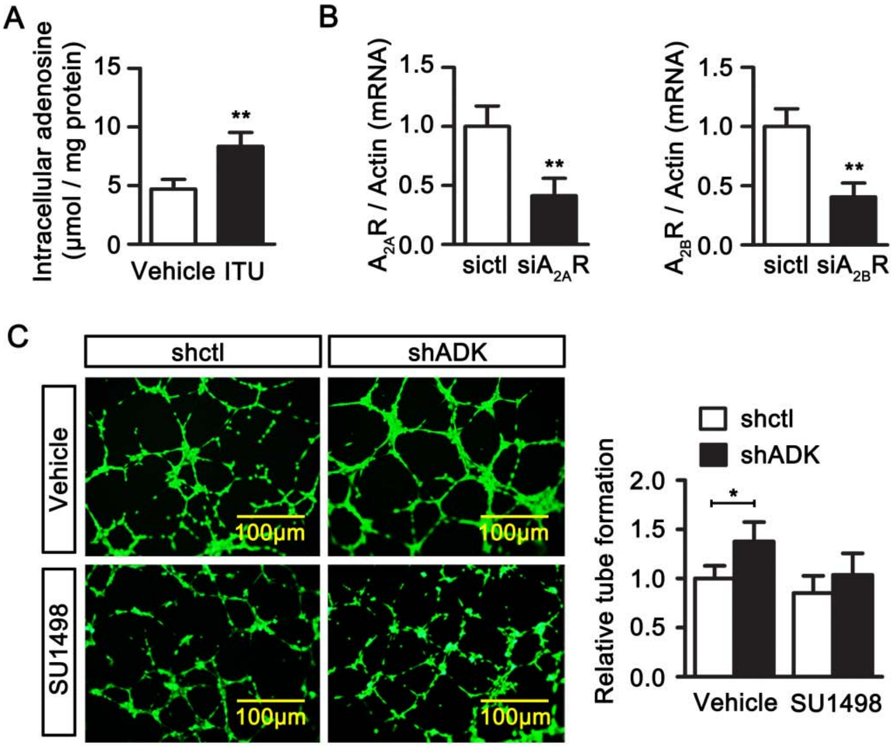

Appendix Figure S5. ADK inhibitor efficiency, adenosine receptor KD efficiency and the effect of VEGFR2 on ADK KD-increased endothelial tube formation.

A, Quantification of intracellular adenosine in vehicle and ITU-treated HUVECs. Cells were treated with vehicle or ITU at 10 µM for 24 hours. Adenosine in collected cell lysates was measured by HPLC. Results are from four independent experiments.

B, Real-time PCR analysis of mRNA levels of $A_{2A}R$ and $A_{2B}R$ in HUVECs transduced with sictl, $siA_{2A}R$ or $siA_{2B}R$. Results are from four independent experiments.

C, Representative images and quantification of capillary network formation of control and ADK KD HUVECs treated with or without VEGFR2 inhibitor SU1498 at 1µM and cultured in growth factor–deprived Matrigel for 16 hours. Results are from four independent experiments.

Data information: For all bar graphs, data are the Mean ± SD, * $P < 0.05$ and ** $P < 0.01$ for indicated comparisons (Unpaired, two-tailed Student's *t*-test for A and B; One-way ANOVA with Tukey's *post-hoc* test for C). The exact *P*-values are specified in Appendix Table S1.

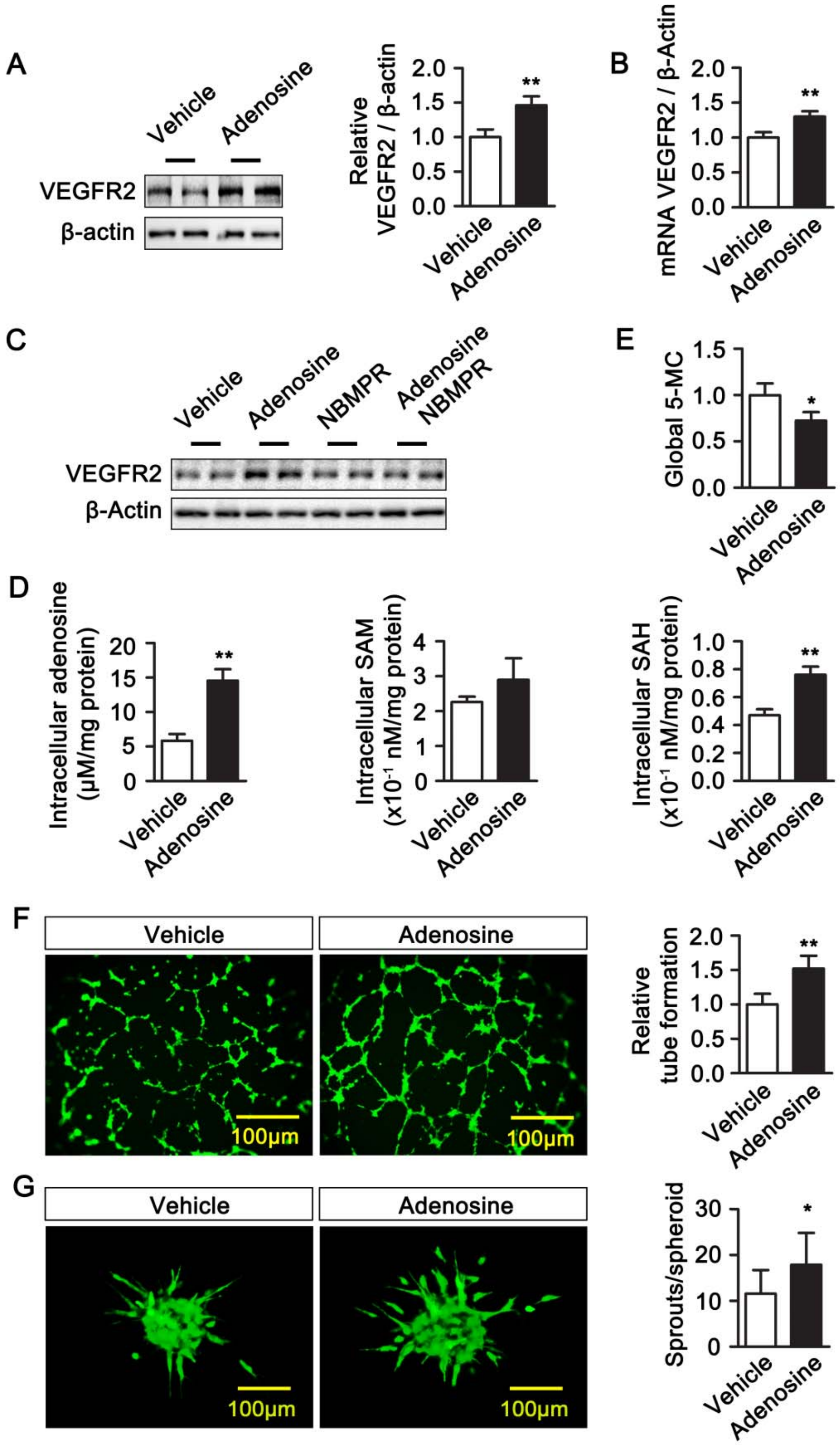

Appendix Figure S6. The effect of exogenous adenosine on DNA methylation, VEGFR2 expression and endothelial functions.

A, Representative images and quantification of Western blot analysis of VEGFR2 protein expression in vehicle and adenosine-treated HUVECs. Results are from three independent experiments.

B, Real-time PCR analysis of VEGFR2 mRNA expression in vehicle and adenosine-treated HUVECs. Results are from three independent experiments.

C, Representative images of Western blot analysis of VEGFR2 protein expression in control and adenosine-treated HUVECs pretreated with vehicle or NBMPR (10 μM). Results are from three independent experiments.

D, Quantification of intracellular adenosine, SAM and SAH in vehicle and adenosine-treated HUVECs. Results are from three independent experiments.

E, Quantification of 5-methylcytosine (5-mC) in vehicle and adenosine-treated HUVECs. Results are from three independent experiments.

For **A**-**E**, cells were treated with vehicle or adenosine at 10 μM for 24 hours. Adenosine treatment was repeated every 6 hours.

F, Representative images and quantification of network formation of vehicle and adenosine-treated HUVECs cultured in growth factor–deprived Matrigel. Results are from three independent experiments.

G, Endothelial sprouting of vehicle and adenosine-treated HUVECs in a spheroid assay (n=10 spheroids per group).

Data information: For all bar graphs, data are the Mean ± SD, * $P < 0.05$ and ** $P < 0.01$ (Unpaired, two-tailed Student's *t*-test). The exact *P*-values are specified in Appendix Table S1.

Source data are available online for this figure.

Appendix Table S1. Statistical analysis information.

Figure	p values	n per group
**1B**	p(0h vs. 6h)=0.0069<br>p(0h vs. 12h)<0.0001<br>p(0h vs. 24h)<0.0001<br>p(0h vs. 48h)<0.0001	n=4; 4; 4; 4; 4; 4
**1D (left)**	p(0h vs. 6h)=0.0002<br>p(0h vs. 12h)<0.0001<br>p(0h vs. 24h)<0.0001<br>p(0h vs. 48h)<0.0001	n=4; 4; 4; 4; 4; 4
**1D (right)**	p(0h vs. 12h)=0.0497<br>p(0h vs. 24h)=0.0010<br>p(0h vs. 48h)<0.0001	n=4; 4; 3; 4; 4; 4
**1E**	p(0h vs. 24h)=0.0004<br>p(0h vs. 48h)<0.0001	n=4; 4; 4;
**2A**	p=0.0003	n=3; 3
**2B**	p=0.0038	n=3; 3
**2C**	p=0.0009	n=3; 3
**2D**	p=0.0393	n=4; 4
**2E**	p=0.0257	n=3; 3
**2F (left)**	p<0.0001	n=30; 30
**2F (right)**	p<0.0001	n=30; 30
**2G**	p(shctl at 72h vs. shADK at 72h)=0.0353<br>p(shctl at 96h vs. shADK at 96h)=0.0046	n=4; 4; 4; 4
**2H**	p(ADK^{WT} at 96h vs. ADK^{VEC-KO} at 96h)=0.0095	n=4; 4 at each time point
**3C**	p=0.0008	n=4; 4
**3D (left)**	p=0.0032	n=4; 4
**3D (right)**	p=0.0065	n=4; 4
**4A**	p=0.0003	n=6; 6
**4C**	p=0.0061	n=6; 6
**4D**	p=0.0128	n=5; 6
**4E**	p=0.0010	n=6; 6
**4F**	p=0.0005	n=6; 6
**4G**	p=0.0048	n=6; 6
**4H**	p=0.0155	n=6; 7
**5B**	p(day3)<0.0001<br>p(day5)<0.0001<br>p(day7)=0.0106	n=7; 7 at each time point
**5C**	p=0.0015	n=7; 7
**5E**	p(D7)<0.0001<br>p(D14)<0.0001	n=7; 7
**5F**	p=0.0014	n=7; 7
**5G**	p(Ischemia)<0.0001	n=7; 7
**6A (left)**	p=0.0001	n=4; 4
**6A (right)**	p<0.0001	n=4; 4
**6B**	p=0.04	n=4; 4

**6C**	p=0.0178	n=4; 4
**6D**	p(Vehicle)=0.0107<br>p(ZM+MRS)=0.0140	n=4; 4; 4; 4
**6H**	p(sictl)=0.0257<br>p(siA2Rs)=0.0231	n=4; 4; 4; 4
**6I**	p=0.0092	n=4; 4
**7A**	p(VEGFR2)=0.00075<br>p(RUNX1)=0.003248<br>p(BRCA1)<0.0001<br>p(NOS3)=0.006961<br>p(GATA4)=0.048421<br>p(SEMA5)=0.001764<br>p(ADDAH1)=0.003823	n=4; 4
**7C**	p<0.0001	n=7; 7
**7G**	p(Vehicle)=0.0027	n=4; 4; 4; 4
**7H**	p(Vehicle)=0.0115	n=10; 10; 10; 10
**8A**	p(Vehicle)=0.0099	n=3; 3; 3; 3
**8B**	p(Vehicle VS. CGS)=0.0112	n=3; 3; 3
**8C**	p(Vehicle)=0.0039309<br>p(ZM)=0.0209101<br>p(MRS)=0.0387039<br>p(ZM+MRS)=0.0346975	n=3; 3
**8D**	p(sictl)=0.0105415<br>p(siA_{2A}R)=0.00406918<br>p(siA_{2B}R)=0.0148207<br>p(siA_2RS)=0.00458093	n=3; 3
**EV1A**	p(Normoxia)<0.0001<br>p(Hypoxia)<0.0001	n=4; 4; 4; 4
**EV1B**	p(Normoxia)<0.0001<br>p(Hypoxia)<0.0001	n=4; 4; 3; 4
**EV1C**	p(sictl+normoxia VS. sictl+hypoxia)=0.0005<br>p(Hypoxia)=0.0001	n=4; 4; 4; 4
**EV1D**	p(siADK+normoxia VS. siADK+hypoxia)=0.0045<br>p(Hypoxia)=0.0405	n=4; 4; 4; 4
**EV1E**	p=0.0004	n=4; 4
**EV1F**	p=0.0013	n=4; 4
**EV1G**	p(Ad-ctl VS. Ad-HIF-1α)<0.0001<br>p(Ad-ctl VS. Ad-HIF-2α)<0.0001	n=4; 4; 4
**EV2A**	p=0.0338	n=3; 3
**EV2B**	p=0.0143	n=3; 3
**EV2C**	p=0.0002	n=30; 30
**S2A**	p(A_1R)=0.019762<br>p(A_{2A}R)<0.0001<br>p(A_{2B}R)=0.000962518<br>p(ADK)<0.0001	n=3; 3
**S2B**	p(Vehicle VS. CGS)=0.0185<br>p(CGS VS. CGS+ZM)=0.0045<br>p(Vehicle VS. NECA)=0.0065	n=3; 3

	p(NECA VS. NECA+MRS)=0.0407	
**S2C**	p(Vehicle)=0.0211<br>p(Antagonists)=0.0312	n=10; 10; 10; 10
**S2D**	p(Vehicle)=0.0097<br>p(Antagonists)=0.0308	n=4; 4; 4; 4
**S3A**	p=0.0021	n=4; 4
**S3B (left)**	p<0.0001	n=4; 4
**S3B (right)**	p<0.0001	n=4; 4
**S3C**	p(Vehicle)=0.0194	n=4; 4; 4; 4
**S4A**	p=0.0093	n=3; 3
**S4B**	p=0.0093	n=3; 3
**S4D (left)**	p=0.0015	n=3; 3
**S4D (right)**	p=0.0023	n=3; 3
**S4E**	p=0.0377	n=3; 3
**S4F**	p=0.0207	n=3; 3
**S4G**	p=0.0177	n=10; 10

Appendix Table S2: Pro-angiogenic genes with increased and decreased methylation upon ADK knockdown.

Gene; name	Accession #	Probes	shADK CH_3 ↑	shADK CH_3 ↓	shADK CH_3 ↑	shADK CH_3 ↓
ACVRL1; Serine/threonine-protein kinase receptor R3	NM_000020	13	2	11	15%	85%
ADM; ADM	NM_001124	17	6	11	35%	65%
ADM2; ADM2	NM_024866	10	2	8	20%	80%
AGGF1; Angiogenic factor with G patch and FHA domains 1	NM_018046	9	1	8	11%	89%
ALOX12; Arachidonate 12-lipoxygenase, 12S-type	NM_000697	11	1	10	9%	91%
ANGPT2; Angiopoietin-2	NM_001118888	7	5	2	71%	29%
ANXA3; Annexin A3	NM_005139	10	2	8	20%	80%
AQP1; Aquaporin-1	NM_198098	14	2	12	14%	86%
BRCA1; Breast cancer type 1 susceptibility protein	NM_007300	37	15	22	41%	59%
BTG1; Protein BTG1	NM_001731	7	1	6	14%	86%
C3; Complement C3	NM_000064	4	0	4	0%	100%
C3AR1; C3a anaphylatoxin chemotactic receptor	NM_004054	5	2	3	40%	60%
C5; Complement C5	NM_001735	1	0	1	0%	100%
C5AR1; C5a anaphylatoxin chemotactic receptor 1	NM_001736	4	2	2	50%	50%
C6; Complement component C6	NM_001115131	5	0	5	0%	100%
CAMP; Cathelicidin antimicrobial peptide	NM_004345	5	3	2	60%	40%
CCBE1; Collagen and calcium-binding EGF domain-containing protein 1	NM_133459	8	1	7	13%	88%
CCL11; Eotaxin	NM_002986	6	1	5	17%	83%
CCL24; C-C motif chemokine 24	NM_002991	7	1	6	14%	86%
CCL5; C-C motif chemokine	NM_002985	7	3	4	43%	57%
CCR2; C-C chemokine receptor type 2	NM_001123041	4	2	2	50%	50%
CCR3; C-C chemokine receptor type 3	NM_001837	7	0	7	0%	100%
CD34; Hematopoietic progenitor cell antigen CD34	NM_001773	8	3	5	38%	63%
CELA1; Chymotrypsin-like elastase family member 1	NM_001971	2	0	2	0%	100%
CHI3L1; Chitinase-3-like protein 1	NM_001276	7	0	7	0%	100%
CHRNA7; Neuronal acetylcholine receptor subunit alpha-7	NM_000746	8	1	7	13%	88%
CIB1; Calcium and integrin-binding	NM_006384	4	0	4	0%	100%

protein 1						
CMA1; Chymase	NM_001836	2	0	2	0%	100%
CTSH; Pro-cathepsin H	NM_004390	4	0	4	0%	100%
CX3CL1; Fractalkine	NM_002996	7	4	3	57%	43%
CX3CR1; CX3C chemokine receptor 1	NM_001171172	6	0	6	0%	100%
CXCR2; C-X-C chemokine receptor type 2	NM_001557	11	2	9	18%	82%
CYP1B1; Cytochrome P450 1B1	NM_000104	28	7	21	25%	75%
CYSLTR2; Cysteinyl leukotriene receptor 2	NM_020377	4	0	4	0%	100%
DDAH1; N(G),N(G)-dimethylarginine dimethylaminohydrolase 1	NM_012137	32	4	28	13%	88%
DLL1; Delta-like protein 1	NM_005618	14	3	11	21%	79%
ECM1; Extracellular matrix protein 1	NM_022664	4	1	3	25%	75%
EPHA1; Ephrin type-A receptor 1	NM_005232	13	3	10	23%	77%
ERAP1; Endoplasmic reticulum aminopeptidase 1	NM_001040458	12	1	11	8%	92%
ETS1; Protein C-ets-1	NM_001143820	9	1	8	11%	89%
F3; Tissue factor	NM_001993	11	2	9	18%	82%
FGF1; Fibroblast growth factor 1	NM_001144935	32	9	23	28%	72%
FGF2; Fibroblast growth factor 2	NM_002006	8	1	7	13%	88%
FLT1; Vascular endothelial growth factor receptor 1	NM_001160030	7	1	6	14%	86%
FOXC2; Forkhead box protein C2	NM_005251	16	4	12	25%	75%
GATA2; Endothelial transcription factor GATA-2	NM_032638	30	12	18	40%	60%
GATA4; Transcription factor GATA-4	NM_002052	31	1	30	3%	97%
GATA6; Transcription factor GATA-6	NM_005257	10	3	7	30%	70%
GREM1; Gremlin-1	NM_013372	12	1	11	8%	92%
HDAC7; Histone deacetylase 7	NM_001098416	7	2	5	29%	71%
HDAC9; Histone deacetylase 9	NM_178423	9	1	8	11%	89%
HGF; Hepatocyte growth factor	NM_001010931	7	0	7	0%	100%
HIF1A; Hypoxia-inducible factor 1-alpha	NM_001530	9	2	7	22%	78%
HIPK1; Homeodomain-interacting protein kinase 1	NM_152696	18	6	12	33%	67%
HIPK2; Homeodomain-interacting protein kinase 2	NM_001113239	2	0	2	0%	100%
HMGB1; High mobility group protein B1	NM_002128	14	2	12	14%	86%
HMOX1; Heme oxygenase 1	NM_002133	5	1	4	20%	80%
HSPB1; Heat shock protein beta-1	NM_001540	12	2	10	17%	83%

HYAL1; Hyaluronidase-1	NM_153285	10	7	3	70%	30%
IL1A; Interleukin-1 alpha	NM_000575	6	0	6	0%	100%
IL1B; Interleukin-1 beta	NM_000576	7	2	5	29%	71%
ISL1; Insulin gene enhancer protein ISL-1	NM_002202	15	1	14	7%	93%
ITGA5; Integrin alpha-5	NM_002205	7	0	7	0%	100%
ITGB2; Integrin beta-2	NM_001127491	22	8	14	36%	64%
JAK1; Tyrosine-protein kinase JAK1	NM_002227	25	3	22	12%	88%
KDR; Vascular endothelial growth factor receptor 2	NM_002253	12	0	12	0%	100%
LRG1; Leucine-rich alpha-2-glycoprotein	NM_052972	4	1	3	25%	75%
MTDH; Protein LYRIC	NM_178812	10	2	8	20%	80%
NODAL; Nodal homolog	NM_018055	10	1	9	10%	90%
NOS3; Nitric oxide synthase, endothelial	NM_000603	12	1	11	8%	92%
NOTCH4; Neurogenic locus notch homolog protein 4	NM_004557	8	2	6	25%	75%
NR2E1; Nuclear receptor subfamily 2 group E member 1	NM_003269	17	4	13	24%	76%
PDCD6; Programmed cell death protein 6	NM_013232	13	1	12	8%	92%
PDCL3; Phosducin-like protein 3	NM_024065	10	1	9	10%	90%
PGF; Placenta growth factor	NM_002632	9	2	7	22%	78%
PIK3R6; Phosphoinositide 3-kinase regulatory subunit 6	NM_001010855	9	0	9	0%	100%
PLCG1; 1-phosphatidylinositol 4,5-bisphosphate phosphodiesterase gamma-1	NM_002660	9	0	9	0%	100%
PPP1R16B; Protein phosphatase 1 regulatory inhibitor subunit 16B	NM_015568	20	1	19	5%	95%
PRKCA; Protein kinase C alpha type	NM_002737	8	3	5	38%	63%
PRKCB; Protein kinase C beta type	NM_002738	7	1	6	14%	86%
PRKD1; Serine/threonine-protein kinase D1	NM_002742	9	2	7	22%	78%
PRKD2; Serine/threonine-protein kinase D2	NM_001079880	13	1	12	8%	92%
PTGIS; Prostacyclin synthase	NM_000961	7	1	6	14%	86%
PTGS2; Prostaglandin G/H synthase 2	NM_000963	11	4	7	36%	64%
PTK2B; Protein-tyrosine kinase 2-beta	NM_173175	21	4	17	19%	81%
RAPGEF3; Rap guanine nucleotide exchange factor 3	NM_006105	17	5	12	29%	71%
RHOB; Rho-related GTP-binding protein RhoB	NM_004040	15	5	10	33%	67%
RLN2; Prorelaxin H2	NM_005059	2	0	2	0%	100%

RRAS; Ras-related protein R-Ras	NM_006270	7	3	4	43%	57%
RUNX1; Runt-related transcription factor 1	NM_001754	20	4	16	20%	80%
SASH1; SAM and SH3 domain-containing protein 1	NM_015278	13	4	9	31%	69%
SEMA5A; Semaphorin-5A	NM_003966	24	2	22	8%	92%
SFRP2; Secreted frizzled-related protein 2	NM_003013	41	8	33	20%	80%
SPHK1; Sphingosine kinase 1	NM_182965	14	3	11	21%	79%
TBXA2R; Thromboxane A2 receptor	NM_201636	14	5	9	36%	64%
TEK; Angiopoietin-1 receptor	NM_000459	3	1	2	33%	67%
TGFBR2; TGF-beta receptor type-2	NM_001024847	16	4	12	25%	75%
TMIGD2; Transmembrane and immunoglobulin domain-containing protein 2	NM_144615	5	2	3	40%	60%
TNFSF12; Tumor necrosis factor ligand superfamily member 12	NM_003809	7	0	7	0%	100%
TWIST1; Twist-related protein 1	NM_000474	31	3	28	10%	90%
UTS2; Urotensin-2	NM_006786	7	1	6	14%	86%
UTS2R; Urotensin-2 receptor	NM_018949	8	4	4	50%	50%
VASH2; Vasohibin-2	NM_001136475	13	1	12	8%	92%
VEGFA; Vascular endothelial growth factor A	NM_001171625	11	2	9	18%	82%
VEGFB; Vascular endothelial growth factor B	NM_003377	6	0	6	0%	100%
VEGFC; Vascular endothelial growth factor C	NM_005429	6	1	5	17%	83%
WNT5A; Protein Wnt-5a	NM_003392	14	3	11	21%	79%
ZC3H12A; Ribonuclease ZC3H12A	NM_025079	14	1	13	7%	93%
ZNF304; Zinc finger protein 304	NM_020657	11	1	10	9%	91%

Appendix Table S2. Pro-angiogenic genes with decreased methylation upon ADK knockdown.

Following the Infinium methylation assay, DNA methylation analysis was carried out on a group of 109 genes related to pro-angiogenic function as defined in the program of Gene Set Enrichment Analysis (GSEA). Column 1: gene symbols and names according to the HUGO Gene Nomenclature Committee; column 2: gene accession number according to the National Center for Biotechnology Information; column 3: number of different probes for each gene represented on the array; column 4: CH_3↑, number of probes with increased methylation in the shADK group compared with the shctl group; column 5: CH_3↓, number of probes with reduced

methylation in the shADK group compared with the shctl group; column 6: CH_3↑, percentage of probes with increased methylation in the shADK group compared with the shctl group; column 7: CH3↓, percentage of probes with reduced methylation in the shADK group compared with the shctl group. The level of DNA methylation in the 14 green highlighted pro-angiogenic genes are considered to be significantly decreased in the shADK group compared with the shctl group.

Appendix Table S3: Anti-angiogenic genes with increased and decreased methylation upon ADK knockdown.

Gene; name	Accession #	Probes	shADK CH_3 ↑	shADK CH_3 ↓	shADK CH_3 ↑	shADK CH_3 ↓
ADRB2; Adrenoceptor beta 2	NM_000024	11	0	11	0%	100%
AGT; Angiotensinogen	NM_000029	7	4	3	57%	43%
ANGPT2; Angiopoietin 2	NM_001118887	7	5	2	71%	29%
ANGPT4; Angiopoietin 4	NM_001322809	7	2	5	29%	71%
ANGPTL4; Angiopoietin-like 4	NM_001039667	9	3	6	33%	67%
APOH; Apolipoprotein H	NM_000042	4	4	0	100%	0%
BAI1; ADGRB1, Adhesion G protein-coupled receptor B1	NM_001702	11	6	5	55%	45%
BAI2; ADGRB2, Adhesion G protein-coupled receptor B2	NM_001294335	21	7	14	33%	67%
BAI3; ADGRB3, Adhesion G protein-coupled receptor B3	NM_001704	15	3	12	20%	80%
CHGA; Chromogranin A	NM_001275	7	2	5	29%	71%
COL4A2; Collagen type IV alpha 2	NM_001846	2	0	2	0%	100%
COL4A3; Collagen type IV alpha 3	NM_000091	11	0	11	0%	100%
CXCL10; C-X-C motif chemokine 10	NM_001565	4	3	1	75%	25%
DAB2IP; DAB-2 interacting protein	NM_032552	4	0	4	0%	100%
DCN; Decorin	NM_001920	9	2	7	22%	78%
DLL4; Delta-like 4	NM_019074	10	3	7	30%	70%
ECSCR; Endothelial cell surface expressed chemotaxis and apoptosis regulator	NM_001077693	4	2	2	50%	50%
FASLG; Fas ligand	NM_000639	5	3	2	60%	40%
FOXC1; Forkhead box C1	NM_001453	13	5	8	38%	62%
GDF2; Growth differentiation factor 2	NM_016204	13	2	11	15%	85%
GHRL; Ghrelin/obestatin prepropeptide	NM_001134941	7	5	2	71%	29%
GPR4; G protein-coupled receptor 4	NM_005282	14	9	5	64%	36%
GTF2I; General transcription factor IIi	NM_001163636	14	7	7	50%	50%
HDAC5; Histone deacetylase 5	NM_001015053	16	11	5	69%	31%
HHEX; Hematopoietically expressed homeobox	NM_002729	7	1	6	14%	86%
HOXA5; Homeobox A5	NM_019102	45	5	40	11%	89%
HRG; Histidine rich glycoprotein	NM_000412	4	3	1	75%	25%
IL17F; Interleukin 17F	NM_052872	6	0	6	0%	100%
ITGB1BP1; Integrin subunit beta 1 binding protein 1	NM_001319066	12	6	6	50%	50%
KLF4; Kruppel-like factor 4	NM_001314052	4	0	4	0%	100%
KLK3; Kallikrein related peptidase 3	NM_001030047	3	3	0	100%	0%
KRIT1; KRIT1, ankyrin repeat containing	NM_001013406	14	4	10	29%	71%
LECT1; Leukocyte cell derived chemotaxin 1	NM_001011705	6	1	5	17%	83%

LIF; Leukemia inhibitory factor	NM_001257135	6	3	3	50%	50%
MAP2K5; Mitogen-activated protein kinase kinase 5	NM_001206804	8	1	7	13%	87%
MEG3; Maternally expressed 3		22	14	8	64%	36%
MMRN2; Multimerin 2	NM_024756	5	2	3	40%	60%
NF1; Neurofibromin 1	NM_001042492	12	4	8	33%	67%
NOTCH1; Notch 1	NM_017617	2	1	1	50%	50%
NPPB; Natriuretic peptide B	NM_002521	11	4	7	36%	74%
NPR1; Natriuretic peptide receptor 1	NM_000906	17	4	13	24%	76%
PDCD10; Programmed cell death 10	NM_007217	13	6	7	46%	54%
PDE3B; Phosphodiesterase 3B	NM_000922	12	4	8	33%	67%
PF4; Platelet factor 4	NM_002619	9	1	8	11%	89%
PML; Promyelocytic leukemia	NM_002675	8	4	4	50%	50%
PTN; Pleiotrophin	NM_001321386	21	14	7	67%	33%
PTPRM; Protein tyrosine phosphatase, receptor type M	NM_001105244	9	1	8	11%	89%
RHOA; Ras homolog family member A	NM_001313941	12	3	9	25%	75%
ROCK1; Rho associated coiled-coil containing protein kinase 1	NM_005406	14	4	10	29%	71%
ROCK2; Rho associated coiled-coil containing protein kinase 2	NM_001321643	7	1	6	14%	86%
SEMA3E; Semaphorin 3E	NM_001178129	8	1	7	13%	87%
SEMA4A; Semaphorin 4A	NM_001193300	6	3	3	50%	50%
SERPINE1; Serpin family E member 1	NM_000602	10	3	7	30%	70%
SERPINF1; Serpin family F member 1	NM_002615	11	6	5	55%	45%
SPARC; Secreted protein acidic and cysteine rich	NM_001309443	11	2	9	18%	82%
SPINK5; Serine peptidase inhibitor, Kazal type 5	NM_001127698	5	3	2	60%	40%
STAB1; Stabilin 1	NM_015136	10	3	7	30%	70%
STARD13; StAR related lipid transfer domain containing 13	NM_001243466	31	11	20	35%	65%
STAT1; Signal transducer and activator of transcription 1	NM_007315	13	5	8	38%	62%
SULF1; Sulfatase 1	NM_001128204	21	6	15	29%	71%
SYNJ2BP; Synaptojanin 2 binding protein	NM_018373	6	3	3	50%	50%
TCF4; Transcription factor 4	NM_001083962	11	4	7	36%	64%
TCF7L2; Transcription factor 7 like 2	NM_001146274	14	5	9	36%	64%
TEK; TEK receptor tyrosine kinase	NM_000459	3	1	2	33%	67%
THBS1; Thrombospondin 1	NM_003246	23	5	18	22%	78%
THBS2; Thrombospondin 2	NM_003247	14	7	7	50%	50%
THBS4; Thrombospondin 4	NM_001306212	9	2	7	22%	78%
TIE1; Tyrosine kinase with immunoglobulin like and EGF like domains 1	NM_001253357	6	1	5	17%	83%

VASH1; Vasohibin 1	NM_014909	13	1	12	8%	92%

Appendix Table S3. Anti-angiogenic genes with decreased methylation upon ADK knockdown.

Following the Infinium methylation assay, DNA methylation analysis was carried out on a group of 69 genes related to anti-angiogenic function as defined in the program of Gene Set Enrichment Analysis (GSEA). Column 1: gene symbols and names according to the HUGO Gene Nomenclature Committee; column 2: gene accession number according to the National Center for Biotechnology Information; column 3: number of different probes for each gene represented on the array; column 4: CH_3↑, number of probes with increased methylation in the shADK group compared with the shctl group; column 5: CH_3↓, number of probes with reduced methylation in the shADK group compared with the shctl group; column 6: CH_3↑, percentage of probes with increased methylation in the shADK group compared with the shctl group; column 7: CH3↓, percentage of probes with reduced methylation in the shADK group compared with the shctl group. The level of DNA methylation in the GDF2 gene highlighted in green is considered to be significantly decreased in the shADK group compared with the shctl group.

Appendix Table S4. Primer sequences for qRT-PCR analysis

Gene	Forward primer (5'-3')	Reverse primer (5'-3')
Human ADK	TGCCCTAATTGCTTCCTGAG	TTGGCATTTAAGTGGCACTATC
Human ENT1	GGCCCAAGAAAGTGAAGCCA	ACCACTCAGGATCACCCCTG
Human ENT2	CTTCCATACCCACTCTCTCACC	GAGAGAGAGGGGATTGGGTC
Human HIF-1α	GAACGTCGAAAAGAAAAGTCTCG	CCTTATCAAGATGCGAACTCACA
Human HIF-2α	CGGAGGTGTTCTATGAGCTGG	AGCTTGTGTGTTCGCAGGAA
Human VEGFR2	TTTGGTTCTGTCTTCCAAAGT	ATGCTCAGCAGGATGGCAA
Human A1R	TGCGAGTTCGAGAAGGTCATC	AGCTGCTTGCGGATTAGGTA
Human A2AR	CGAGGGCTAAGGGCATCATTG	CTCCTTTGGCTGACCGCAGTT
Human A2BR	CTCTTCCTCGCCTGCTTCGTG	TTATACCTGAGCGGGACACAG
Human A3R	TACATCATTCGGAACAAACTC	GTCTTGAACTCCCGTCCATAA
Human Runx1	CTGCTCCGTGCTGCCTAC	AGCCATCACAGTGACCAGAGT
Human BRCA1	AACCCCTTACCTGGAATCTG	TCCCTGCTCAGACTTTCTTC
Human ALOX12	GATGATCTACCTCCAAATATG	CTGGCCCCAGAAGATCTG
Human Sema5A	GAACCGGAAGCGTGTT	CAGTGAGATGTGGGTTGAAG
Human DDAH1	GCAACTTTAGATGGCGGAGA	CCAGTTCAGACATGCTCACG
Human CCR3	ATGCTGGTGACAGAGGTGAT	AGGTGAGTGTGGAAGGCTTA
Human IL1B	AAACCTCTTCGAGGCACAAG	GTTTAGGGCCATCAGCTTCA
Human PTK2B	GGTGCAATGGAGCGAGTATT	GCCAGTGAACCTCCTCTGA
Human GATA4	TCAAATTGGGATTTTCCGGA	GCACGTAGACTGGCGAGGA
Human PDCD6	ATGGCCGCCTACTCTTACCG	AGAGGCCAGGGTCATACGATAC
Human GDF2	CTGCCCTTCTTTGTTGTCTT	CCTTACACTCGTAGGCTTCATA
Human NOS3	AGGAACCTGTGTGACCCTCA	CGAGGTGGTCCGGGTATCC
Human C3	TCGGATGACAAGGTCACCCT	GACAACCATGCTCTCGGTGA
Human CCR2	GACAAGCCACAAGCTGAACA	GAGCCCACAATGGGAGAGTA

Human β-actin	CGAGGCCCAGAGCAAGAGAG	CTCGTAGATGGGCACAGTGTG

Appendix Supplementary Methods

Mouse generation and breeding

The use of experimental animals was approved by the IACUC at the Augusta University in accordance with NIH guidelines. The floxed ADK (ADK$^{flox/flox}$) mice were generated by Xenogen Biosciences Corporation (Cranbury, NJ, USA). Briefly, a BAC clone containing the designated mouse ADK gene was first isolated, confirmed and characterized. A conditional gene targeting construct (2loxP construct), as shown in Fig 3A, was generated. ES cells were transfected with the targeting construct DNA for the gene target in the presence of G418. After screening, G418-resistant clones were analyzed with independent Southern blot analysis with 5', 3' and Neo probes in order to confirm homologous recombination clones. The confirmed ES clones were injected into up to a total of 200 blastocysts. The blastocysts were then injected into pseudo-pregnant females to generate chimeras. Chimeric mice were bred with wild-type mice, and the F1 generation with germline transmission of the mutant allele was confirmed through tail DNA PCR and/or Southern blot analysis. Cell-specific inactivation of ADK (ADK^{VEC-KO}) in endothelial cells was achieved by cross-breeding Cdh5-Cre transgenic mice (The Jackson Laboratory) with ADK$^{flox/flox}$ mice.

Isolation and culture of primary mouse aortic endothelial cells (MAECs)

MAECs were isolated using a previously described method except that Matrigel was replaced with collagen gel (Wang et al, 2016). Collagen gel was prepared as follows. Commercial type I collagen (BD Bioscience, San Jose, CA, USA) was diluted with endothelial growth medium 2 (EGM-2; Lonza, Basel, Switzerland) to a final concentration of 1.75 mg/mL.

0.5 ml collagen gel was added to each well of a 24-well plate, and the collagen gel was then solidified at 37°C for at least 30 min.

Aortic ring preparation: Four-week-old male mice were used. Briefly, mice were sacrificed by CO_2 asphyxiation and cleaned with 70% ethanol. The abdominal and thoracic cavities were opened, and each mouse was perfused with 3 mL PBS via the left ventricle. After removal of perivascular fat and adventitia from the ventral side of the aortas, the aortas were dissected out, rinsed 5 times with fresh PBS, and placed in a sterile dish of cold PBS. The aortas were then cut into ~1 mm length rings, and each aortic ring was opened and laid on the surface of collagen gel with the endothelium directly facing the gel.

Following tissue placement for 36 hours, the gel and aortic piece were kept hydrated with EGM-2. During the above procedures, care was taken to avoid submerging and dislodging the aorta piece from the gel. Explants were then cultured in an incubator at 37°C and 5% CO_2 and monitored daily. For the experiments on endothelial migration, the gels and aortic pieces were processed at different time points based on the protocols. After visible cellular outgrowth from the aortic segments, the medium was aspirated, and the aortic segments were removed in a sterile fashion. The collagen gel was digested by 0.3% collagenase H solution in PBS, and the MAECs were collected.

Isolation and culture of murine lung and heart endothelial cells

Lungs and hearts were minced and placed in DMEM containing 2 mg/ml collagenase (Roche, Basel, Switzerland) for 1 h at 37°C. The digested tissues were separated into single cell suspension using 18-gauge and 20-gauge needles and then passed through a 70 µm cell strainer. The cell suspension was centrifuged and the cell pellets were washed once with washing buffer

(PBS-0.5% BSA supplemented with 2mM EDTA) and resuspended with 90 µl binding buffer per 10^7 total cells, followed by the addition of 10 µl CD31 MicroBeads (130-097-418, Miltenyi Biotec). After incubation at 4°C for 15 min, positive selection was performed by passing the cell suspension through magnetic LS columns (130-042-401, Miltenyi Biotec). The $CD31^+$ endothelial cells were then collected.

Cell culture and treatments

HUVECs (ATCC, Manassas, VA, USA), at a passage of 3-8, and MAECs, at a passage of 2-4, were cultured in EGM-2 (Lonza, Basel, Switzerland). In some experiments, 10 µM 5-iodotubercidin (ITU, Tocris Bioscience, Bristol, United Kingdom), 10 µM adenosine (Sigma, St. Louis, MO, USA), 1 µM SU1498 (Sigma, St. Louis, MO, USA), 5 µM CGS21680 (Tocris Bioscience, Bristol, United Kingdom), 5 µM NECA (Tocris Bioscience, Bristol, United Kingdom), 5 µM MRS1754 (Tocris Bioscience, Bristol, United Kingdom), 5 µM ZM241385 (Tocris Bioscience, Bristol, United Kingdom), 10 µM NBMPR (Tocris Bioscience, Bristol, United Kingdom), 2 µM 5-Aza-2'-deoxycytidine (5-Aza, Sigma, St. Louis, MO, USA), 100 ng/ml hVEGF (R & D systems, Minneapolis, MN, USA), or 100ng/ml mVEGF (R & D systems, Minneapolis, MN, USA) was added to the culture medium.

Adenoviral transduction of HUVECs

The GFP-labeled-ADK shRNA adenovirus targeting the 3' UTR sequence of human ADK and the control adenovirus were constructed by Vector Biolabs (Malvern, PA, USA). Ad-mutHIF-1α encoding the mutant human HIF-1α construct containing mutations at P564A and N803A and Ad-mutHIF-2α containing mutations at P531A and N847A were generated as

previously described (Ahmad et al, 2009). These adenoviruses were expanded inside HEK293 cells, and the virus concentration was determined using an Adeno-XTM rapid titer kit (Clontech, Mountain View, USA). HUVECs at 80% confluence were transduced with the adenovirus (10 pfu/cell) and were used for experiments 36 h after the transduction.

RNA interference

HUVECs were transfected at 60-70 % confluence with 30 nM siRNAs targeting human $A_{2A}R$ (si$A_{2A}R$, Santa Cruz Biotechnology, Dallas, Texas, USA) or targeting human $A_{2B}R$ (si$A_{2B}R$, Santa Cruz Biotechnology) using siRNA transfection reagent (Santa Cruz Biotechnology) per the manufacturer's protocol. Knockdown of HIF-1α or HIF-2α in HUVECs was performed using predesigned SmartPool siRNA (siHIF-1α and siHIF-2α) purchased from Dharmacon (Lafayette, CO, USA). Twenty-four hours after transfection, cells were cultured in fresh complete EGM-2 for an additional 24 hours before further experiments.

Cell number analysis

For cell counting, HUVECs or MAECs were seeded in 6-well plates in triplicate at an equal density, and cell numbers were manually counted at the indicated days with a hemocytometer.

Quantitative real time RT-PCR (qRT-PCR) analysis

The total RNA from HUVECs was extracted with an RNeasy Mini Kit (Qiagen), and qRT-PCR was done as described previously. Briefly, a 0.5-1 μg sample of RNA was utilized as a template for reverse transcription using the iScriptTM cDNA synthesis kit (Bio-Rad). qRT-PCR

was performed on an ABI 7500 Real Time PCR System (Applied Biosystems) with the respective gene-specific primers listed in Appendix Table S4. All samples were amplified in duplicate, and every experiment was repeated independently at least three times. For experiments under normoxia, relative gene expression was calculated using the $2^{-\triangle\triangle ct}$ method against the internal control β-actin. For experiments subjected to hypoxia, relative gene expression was calculated using the $2^{-\triangle\triangle ct}$ method against the internal control RPLP0.

Protein extraction and Western blotting

HUVECs or MAECs were lysed with RIPA buffer (Sigma, St. Louis, MO, USA) with 1% proteinase inhibitor cocktail (Roche, Basel, Switzerland) and 1% PMSF. After centrifugation of the cell lysates, the protein was quantified with a BCA assay and then loaded in a 6-9% SDS-PAGE gel at 10 μg per lane. Antibodies used in this study against the following proteins were as follows: ADK (Abcam, Cambridge, MA, USA; rabbit, 1:1000), p-VEGFR2 (rabbit, 1:1000; Cell Signaling Technology, Danvers, MA, USA), VEGFR2 (rabbit, 1:1000; Cell Signaling Technology, Danvers, MA, USA), p-AKT (rabbit, 1:2000; Cell Signaling Technology, Danvers, MA, USA), AKT (rabbit, 1:2000; Cell Signaling Technology, Danvers, MA, USA), p-p70S6K (rabbit, 1:1000; Cell Signaling Technology, Danvers, MA, USA), p70S6K (rabbit, 1:1000; Cell Signaling Technology, Danvers, MA, USA), p-ERK (rabbit, 1:1000; Cell Signaling Technology, Danvers, MA, USA), ERK (rabbit, 1:1000; Cell Signaling Technology, Danvers, MA, USA), PCNA (rabbit, 1:1000; Cell Signaling Technology, Danvers, MA, USA) and β-actin (rabbit, 1:5000; Cell Signaling Technology, Danvers, MA, USA). Images were taken with the ChemiDoc MP system (Bio-Rad, Hercules, CA, USA), and band densities were quantified using Image Lab software (Bio-Rad, Hercules, CA, USA).

In vitro tube formation analysis

HUVECs were transfected with an ADK shRNA-containing adenovirus or control virus 36 h before the assays were performed, or cells were treated with 10 µM adenosine 48 h before assays were performed. To examine tube formation, growth factor-reduced Matrigel (BD Bioscience, San Jose, CA, USA) was placed in 96-well tissue culture plates (60µl/well) and allowed to form a gel at 37°C for at least 30 min. HUVECs infected with virus were re-suspended in 0.5% FCS growth medium at a 1×10^5 concentration. The adenosine-treated HUVECs were resuspended in 0.5% FCS growth medium supplemented with 10µM adenosine. Aliquots of 150 µl of the cell suspension were added to each well, and the plates were incubated 8 h at 37°C. The endothelial tubes were observed using an inverted fluorescent microscope after staining with Calcein AM (Life Technologies, Grand Island, NY, USA). Three random fields were selected and photographed. Tube formation was analyzed with WimTube quantitative tube formation image analysis program (Ibidi, Martinsried, Germany). The results are expressed as the mean fold-change of tube length compared with the control.

Assay of endothelial cell chemotactic migration

HUVECs were transfected with an ADK shRNA-containing adenovirus or control virus 36 h prior to performing the migration assay. The chemotactic migration of HUVECs was assayed using a transwell chamber equipped with a 6.5-mm-diameter polycarbonate filter with 8 µm pore size. Briefly, 50 ng/ml of recombinant VEGF in endothelial basal medium (Lonza, Basel, Switzerland) containing 1% FCS was placed in the lower wells, and HUVECs suspended in endothelial basal medium containing 1% FCS at a final concentration of 5×10^4 cells/ml were

placed in the upper wells. The chambers were incubated at 37°C for 12 h. Non-migrating cells on the upper surfaces of filters were removed by wiping with a cotton swab, and migrated cells on the back of the filter were fixed and stained with a 1% crystal violet solution (Sigma, St. Louis, MO, USA). Migrated cells on the lower side of the filters were counted at ×200. Eight random fields were counted for every transwell.

BrdU incorporation analysis

HUVECs were synchronized in G0/G1 by total FBS depletion for 12 hours and then treated with 10 μM BrdU (5-bromodeoxyuridine, Sigma, St. Louis, MO, USA) and further incubated with complete growth medium for an additional 24 hours. Following BrdU treatment, cells were fixed, heated at 98°C for 8 min in citric acid buffer for antigen retrieval and incubated with a monoclonal anti-BrdU antibody (mouse, 1:200; Sigma, St. Louis, MO, USA) followed by incubation with an Alexa Fluor 594-labeled anti-mouse secondary antibody (Molecular Probes; 1:250). The cells were then immersed in ProLong Gold mounting medium with DAPI (Invitrogen, Grand Island, USA) to visualize the nuclei. Images were obtained using a Zeiss Axio Observer Z1 inverted microscope at 10 X magnification.

Fibrin gel bead assay

Fibrin gel bead assay was performed as described previously (Nakatsu et al, 2007). Briefly, HUVECs were incubated with Cytodex3 microbeads (Amersham Pharmacia Biotech) at a concentration of 400 cells per bead in 1.5 mL of EGM-2 medium for 4 hours at 37°C. Overnight, cell-coated beads were washed with EGM-2 and then resuspended in 2 mg/mL of fibrinogen solution (Sigma-Aldrich) plus 0.15 U/mL of aprotinin (Sigma-Aldrich) at a concentration of 500

cell-coated beads/mL. Next, 500 μL of fibrinogen/bead suspension was added to the wells of a 24-well plate containing 0.625 units of thrombin (Sigma-Aldrich). Once the gel clotted, 1 ml of EGM2 containing 20,000 fibroblasts was placed on the top of the fibrin gel, and the culture medium was changed every other day. Images were captured and data were quantified between days 5 and 6 by the live-culture imaging system of a Zeiss LSM 780 Inverted Confocal Microscope using a bright field. The number of sprouts and cumulative length of sprouts per bead were quantified from 30 beads per condition.

Spheroid capillary sprouting assay

HUVECs (750 cells) were incubated for 24 hours in 25% EBM-2 (25% EBM-2 complete medium plus 75% EGM-2 medium) supplemented with 0.25% (w/v) methylcellulose (Sigma-Aldrich) to form spheroids as described previously (Heiss et al, 2015). The spheroids were harvested and embedded in 0.9 ml collagen solution in a pre-warmed 24-well plate, with a final concentration of rat type I collagen (BD Biosciences) at 1.5 mg/ml. The spheroid-containing gels were rapidly transferred into a humidified incubator (37 °C, 5 % CO2) and allowed to polymerize for 20 min, after which 0.1 ml EBM-2 basal medium supplemented with 20ng/ml hVEGF (R & D Systems, Minneapolis, MN, USA) and the corresponding compounds were pipetted on top of the gel. After 24 hours, gels were fixed with pre-warmed 4 % paraformaldehyde (PFA), and images were captured with a Zeiss LSM 780 Inverted Confocal Microscope. The number of sprouts per spheroid was quantified from 10 spheroids for each condition using Image J software.

Aortic ring assay

Aortas were harvested from 4-week-old male ADK^{VEC-KO} and control ADK^{WT} mice. Plates (24-well) were coated with collagen gel, and after it had gelled, the rings were placed into the wells. Thirty-six hours later, 1 mL EGM-2 was added to each well. On day 3 or 5, the tissue and the sprouting endothelial cells were fixed and stained with isolectin B4. Endothelial sprouts were time-lapse imaged on an Olympus IX81-ZDC inverted fluorescence microscope (Olympus, Center Valley, PA, USA). Vessel sprouting was quantified by counting the number of vascular sprouts that directly originated from a mouse aorta. The photographed image was divided into 5 regions and sprouts in each region were scored from 0 (least positive) to 1 (most positive) in a double-blind manner. The scores from 5 regions were combined, and the total was scored from 0 (least positive) to 5 (most positive). Each data point was assayed in quintuple.

Analysis of mouse retinal vasculature

For retinal vessel analysis, P4 or P12 pups were given lethal doses of ketamine (Boehringer, Ingelheim am Rhein, Germany), and the eyes were collected and fixed in 4% paraformaldehyde (PFA) for 10 min at 4°C. The retinas were isolated and fixed in PFA for an additional 30 min and then stained overnight at 25°C with Alexa Fluor 594-labeled Isolectin B4 (Molecular Probes, Grand Island, NY, USA) in 1 mM $CaCl_2$ in PBS. Following 2 h of washes, retinas were whole-mounted onto Superfrost/Plus microscope slides (Fisher Scientific, Pittsburgh, PA, USA) with the photoreceptor side down and embedded in SlowFade Antifade reagent (Invitrogen, Grand Island, NY, USA). Images were taken on an Olympus IX81-ZDC inverted fluorescence microscope and confocal fluorescent microscope (LSM 510 META; Carl Zeiss, Jena, Germany). Vessel sprouts were evaluated using a described method (Lobov et al, 2007).

Analysis of embryonic hindbrain angiogenesis

Mouse embryos were harvested at E10.5-11.5 and hindbrain tissue was isolated, stained and analyzed as previously described (Fantin et al, 2013).

Wound healing assay and analysis

A previously described method was used (Lanahan et al, 2014). Briefly, 8-week-old male ADK^{VEC-KO} and control ADK^{WT} mice were anesthetized by intraperitoneal injection of a ketamine (100 mg/kg)/xylazine (10 mg/kg) solution. Wounds were generated with a sterile 6-mm biopsy punch (Miltex Inc, PA) on the mouse back skin without injuring the underlying muscle. Wound regions were photographed using a Leica M125 microscope with an HC80 HD camera (Leica, Wetzlar, Germany) on days 0, 1, 3, 5 and 7. The wound area was calculated using NIH Image J software in a blinded fashion with regard to the animal groups, and the sizes at different time points were expressed as a percentage of the wound area on day 0. Blood perfusion images of the wounds on day 2 of wound generation were obtained by laser speckle contrast imaging. Briefly, the mice were anesthetized using isoflurane, and body temperature was maintained at 37 ±0.2 °C using a thermo-regulated heating pad (Harvard Apparatus, Holliston, MA, United States). Blood perfusion images were acquired using PeriCam PSI HD system (Perimed Inc., Sweden) with a 70 mW built-in laser diode for illumination and a 1388 x 61038 pixel CCD camera for image acquisition. Once the perfusion was consistent, images (2 per sec) were acquired for 2 min at a speed of 2 Hz. Acquired images were analyzed for blood flow using PIMSoft, a PeriCam dedicated computer program (Perimed Inc., Sweden). The mean wound blood flow was calculated as the ratio of the blood flow in the wound area to that of the surrounding normal tissue area and was presented as a percent. Six mice were analyzed in each group.

Hind limb ischemia model

As described previously (Limbourg et al, 2009), the femoral arteries of male ADK^{VEC-KO} and control ADK^{WT} mice at 8 weeks of age were ligated at 2 positions spaced 5 mm apart, and the arterial segment between the ligatures was excised. In a blinded fashion with regard to the animal groups, tissue perfusion was assessed preoperatively, immediately, and 3, 7, and 14 days after surgery. Blood flow images of the foot were acquired by laser speckle contrast imaging (Perimed Inc., Sweden) at 37 ± 0.2 °C under isoflurane anesthesia. Data were analyzed with PIMSoft, a PeriCam-dedicated computer program (Perimed Inc., Sweden) and reported as the ratio of blood flow in the left/right (L/R) hind limb after background subtraction.

Histology and immunohistochemistry

On the day indicated after femoral artery ligation, gastrocnemius muscles were dissected, fixed in 4% PFA for 24 h, dehydrated, embedded in paraffin, sectioned at 7 μm thicknesses, and stained with haematoxylin and eosin (H&E). Areas of necrotic muscle fibers were identified with morphology, differential eosin staining, and infiltrating leukocytes near the degenerating fibers on the whole section at × 200. Inflammation status was scored based on the number of infiltrated leukocytes.

For immunofluorescent staining of CD31 and VEGFR2 in gastrocnemius muscles, following deparaffinization, rehydration and antigen retrieval, sections were blocked and incubated overnight at 4°C with primary antibodies followed by fluorescent secondary antibody for 1 h at room temperature. The capillaries were visualized by fluorescent staining with antibodies against CD31 (rat, 1:200, Dianova, Hamburg, Germany) and VEGFR2 (rabbit, 1:200;

Cell Signaling Technology, Danvers, MA, USA) as well as their respective fluorophore-conjugated secondary antibodies (1:1000; Molecular Probes, Grand Island, NY, USA). The slides were coverslipped using Dapi Vectashield mounting medium (Vector Laboratories, Burlingame, CA, USA). The numbers of CD31- and VEGFR2-positive cells were counted in 5 random high-power fields (magnification x 200 or x 400) with 4 mice for each group, respectively.

For immunostaining for VEGFR2, aortas were fixed in 4% ice-cold PFA overnight, then embedded in paraffin. Slides were deparaffinized, rehydrated, incubated for 30 minutes in diluted normal blocking serum, and then incubated with primary VEGFR2 antibody (rabbit, 1:200; Cell Signaling Technology, Danvers, MA, USA). Sections were then stained with secondary antibody and HRP-DAB detection reagents (brown) and counterstained with haematoxylin (blue).

Global DNA methylation assay

Total genomic DNA was isolated from HUVECs using a DNeasy Blood and Tissue Kit (QIAGEN, Venlo, Netherlands). Global DNA methylation status was assessed using the MethylFlash Methylated DNA quantification kit (Epigentek, Farmingdale, NY, USA) per the manufacturer's instructions.

DNMT activity assay

Nuclear proteins were isolated from adenosine-treated HUVECs using an EpiQuik Nuclear Extraction Kit I per the manufacturer's instruction (Epigentek, Farmingdale, NY, USA). DNMT activity of freshly isolated nuclear proteins was quantified using a fluorimetric EpiQuick

DNMT Activity Assay Ultra kit per the manufacturer's instruction (Epigentek, Farmingdale, NY, USA).

Methylated DNA immunoprecipitation-qRT-PCR

Methylated DNA immunoprecipitation (MeDIP)-qPCR assay was carried out with a Magnetic Methylated DNA Immunoprecipitation kit (Diagenode, Denville, NJ, USA). Briefly, the ADK KD or control HUVECs were collected and lysed using GenDNA Digestion buffer supplemented with GenDNA proteinase K. The genomic DNA was extracted by phenol/chloroform/isoamyl alcohol and then precipitated by 100% ethanol. To gain fragments of 300 +/- 200 bp size, DNA was sheared using the sonicator XL2000 (Misonix, Farmingdale, NY, USA). Conditions were 10 cycles of 20sec ON/70sec OFF at power level 5. Efficiency of shearing was checked by electrophoresis on an agarose gel. The sonicated DNA was then immunoprecipitated by incubating with the 5-methylcytosine antibody and Magbeads. A portion of sheared DNA was kept as input control. Immune complex-bound beads were washed with washing buffers, and then the proteinase K digestion-recovered DNA was checked for the efficiency of methyl DNA immunoprecipitation of the VEGFR2 promoter region by qPCR using VEGFR2-specific primers with the sequences TGGTCGCTGCGTTTCCTC (forward) and CGGGAGCCGGTTCTTTC (reverse). The MeDIP-qPCR data were analyzed the using [delta]Ct method in which the immunoprecipitated sample Ct value was normalized with the input DNA Ct value, and the percentage of precipitation was calculated using the following formula (%Input = 2^-[Ct (meDNA-IP)-Ct (Input)] x dilution factor x 100%).

Infinium methylation assay

The bisulfite conversion of genomic DNA was conducted using an EZ DNA Methylation Gold kit (Zymo Research). 200ng of converted DNA was analyzed using an Illumina Infinium 450K Methylation array according to the manufacturer's protocols (Illumina). The quantitative value of DNA methylation was calculated from the ratio of fluorescent signals from the methylated alleles to the sum of the signals from the methylated and unmethylated alleles, assigned via the Genome Studio Methylation Software Module (Illumina). Minfi package in Bioconductor (http://bioconductor.org/packages /release/bioc/html/minfi.html) was used for quality control and normalization before statistical analysis. The average of the DNA methylation level across all the CpG sites within a sample was used as an index of the global DNA methylation level of the sample. Student *t*-test analysis from Limma package was used to identify differentially methylated CpG sites between the control shctl group and the shADK group in the methylation array data. Raw *P*-values were converted to false discovery rates (FDRs) based on Benjamini and Hochberg. A FDR < 0.2 was used as the cut-off value to select differentially methylated CpG sites in the genes in the pathway of positive regulation of angiogenesis (n=3, obtained from the AmiGO2).

DNA bisulfite sequencing analysis

To confirm the methylation status of the VEGFR2 promoters, HUVEC genomic DNA was bisulfite converted using an EpiTect Bisulfite Kit (QIAGEN, Venlo, Netherlands) according to the manufacturer's instructions. After bisulfite conversion, the VEGFR2 promoter region was amplified using the following primers (product size, 612bp): forward, 5'-AAG TTG TTG TTT TGG GAT GTT T-3'; reverse, 5'-AAA TAA ACT CCT TAC CCA CAA A-3'. PCR amplifications were carried out at 95°C for 10 minutes, followed by 35 cycles of 95°C for 30

seconds, 54.7°C for 30 seconds and 72°C for 1 minute, with final extension at 72°C for 20 minutes. The resulting PCR products were first checked by agarose gel electrophoresis and then subcloned into a TA vector using a TOPO® Cloning Kit (Invitrogen, Carlsbad, CA). Ten positive clones originating from each PCR product were randomly selected for DNA sequencing analysis with T3 primer in an ABI 310 automated sequencing system (ACGT Inc, Wheeling, IL). DNA sequencing results were analyzed by Sequence Scanner Software 2.0 (Applied Biosystems, Grand Island, NY) and BiQ analyzer 2.0 (http://biq-analyzer.bioinf.mpi-sb.mpg.de).

DNA extraction and methylation-specific PCR (MSP)

DNA was extracted from MAECs using a DNeasy Blood and Tissue Kit (QIAGEN, Venlo, Netherlands). Methylation status was determined by methylation-specific PCR using a methylation kit (EpiTect Bisulfite Kit; QIAGEN, Venlo, Netherlands). MethPrimer software was used for prediction of the CpG island of VEGFR2 (NM_010612.2) and design of methylation-specific primers. The sequences of primers for methylated VEGFR2 at the promoter region were TGTTTTTAGATGCGATTTGTCGTTC (forward) and AAAATAAAAACTCCCTACGTCCGAC (reverse); for unmethylated VEGFR2 promoter, TTTTTAGATGTGATTTGTTGTTTGG (forward) and AAAATAAAAACTCCCTACATCCAAC (reverse). The PCR conditions were 94°C for 2 minutes with hot start, and then 94°C for 30 seconds, 58°C and 60°C for 30 seconds, and 72°C for 1 min, repeated for 35 cycles. The template DNA for a positive control was mouse genomic DNA that was enzymatically methylated at all CpGs by M.SssI methyltransferase 1 (Thermo Scientific, Grand Island, NY, USA). Template DNA for a negative control was a fragment of unmethylated mouse DNA amplified with a pair of primers (forward:

ACACAGCTTACTCTCTTGGG; reverse: ACTGACCAACTCACTGCAAG). The subsequent 485 bp product contains a MSP-amplified DNA fragment. Image J was used for semiquantitative measurement of methylated and unmethylated VEGFR2. Methylated VEGFR2 was normalized by comparison with unmethylated VEGFR2. The experiments were repeated three times.

Measurement of intracellular cAMP

The cAMP concentrations were determined using a Cyclic AMP XP® assay kit (Cell Signaling Technology, Danvers, MA, USA) according to the manufacturer's protocol. Briefly, HUVECs were seeded at a density of 1×10^4 cells/well in a 96-well plate and incubated overnight. Following exposure to the indicated chemicals, the cells were washed with ice-cold PBS and lysed with ice-cold 1× lysis buffer. The HRP-linked cAMP solution was added to the assay plate, incubated at room temperature for 3 h, followed by 4 washes with the 1× wash buffer. The TMB substrate was then added and the absorbance was measured at 450 nm. The cAMP concentration of the sample was calculated using a cAMP standard curve.

Videomicroscopic assessment of arteriolar responsiveness

Videomicroscopy of isolated skeletal muscle (m. gracilis) arterioles were performed as previously described (Bagi et al, 2005). Briefly, arterioles (diameter: ~ 100 µm) were dissected from the m. gracilis obtained from $ADK^{f/f}$ (n=5) and $ADK^{f/f}/Cdh5^{Cre}$ mice (n=5). Isolated arterioles were cannulated at both ends and pressurized (70 mmHg) with the use of hydrostatic pressure reservoirs. Changes in arteriolar diameter were measured with a videocaliper (Colorado Instruments, Colorado Springs, CO, USA) in response to cumulative concentrations of the

endothelium-dependent vasodilator acetylcholine (ACh, 10^{-10} -10^{-6} M) or to the potent vasoconstrictor, serotonin (5-HT, 10^{-10} -10^{-6} M).

Measurement of intracellular adenosine, SAM and SAH levels

Adenosine was measured with reversed-phase HPLC. Briefly, supernatants of HUVEC lysates with protein concentration at 1 mg/mL were separated with a C_{18} reversed-phase analytical column (250 mm x 4.6 mm I.D., 5 μm particle) (Aglient, Santa Clara, CA, USA). The mobile phase consisted of two solvents. Solvent A is a solution of 0.031M $Na_2HPO_4 \cdot 2H_2O$ and 0.068M $NaH_2PO4 \cdot 2H_2O$ adjusted to pH 6.3. Solvent B is a solution of 100% methanol. Solvent A was filtered through a 0.2-μm membrane filter prior to use in the assay. To detect adenosine, the HPLC column was first equilibrated with 80% Solvent A and 20% Solvent B and was held constant at the equilibration conditions for 10min. The flow-rate was 1 mL/min, and detection was monitored at 254 nm. A 20-μl aliquot of the acid extract was applied directly onto the HPLC column. The identity of adenosine was determined by comparing retention times to an adenosine standard and was further confirmed by enzymatic peak shift analysis. The results (μm/mg) indicate the adenosine concentrations (μmol/L) in the cell lysates with protein concentration at 1 mg/mL.

SAH and SAM were measured with reversed-phase HPLC. Briefly, supernatants of HUVEC lysates were separated with a C_{18} reversed-phase analytical column (250 mm x 4.6 mm I.D., 5 μm particle) (Aglient, Santa Clara, CA, USA). The mobile phase consisted of two solvents. Solvent A is a solution of 8 mM octane sulfonic acid sodium salt and 50 mM NaH_2 PO_4 adjusted to pH 3.0 with H_3 PO_4. Solvent B is a solution of 100% methanol. Solvent A was filtered through a 0.2-mm membrane filter before use in the assay. The HPLC column was first

equilibrated with 80% Solvent A and 20% Solvent B and then injected with sample. Separation was obtained using a step gradient with 8 min at the equilibration conditions, 30 s to increase Solvent B to 40%, 12.5 min at the new condition, and 30 s to return to the equilibration conditions. There was a minimum of 10 min before a subsequent injection. The flow-rate was 1 ml/min, and detection was monitored at 254 nm. A 25-µl aliquot of the acid extract was applied directly onto the HPLC column. SAM and SAH were identified based on their retention times and co-chromatography of SAM and SAH standards. Quantification was based on integration of peak areas and compared to the standard calibration curves of SAM and SAH.

Statistical analysis

The data are presented as the mean ± SD and were analyzed by one-way ANOVA followed by Tukey's post-hoc test or Student's *t*-test to evaluate two-tailed levels of significance. Two-way repeated-measures ANOVA with Bonferroni's post-hoc test was done to assess wound size and perfusion improvement over time within groups. The null hypothesis was rejected at $P \leq 0.05$.

Reference

Ahmad A, Ahmad S, Glover L, Miller SM, Shannon JM, Guo X, Franklin WA, Bridges JP, Schaack JB, Colgan SP et al (2009) Adenosine A2A receptor is a unique angiogenic target of HIF-2alpha in pulmonary endothelial cells. Proceedings of the National Academy of Sciences of the United States of America 106: 10684-10689

Bagi Z, Erdei N, Toth A, Li W, Hintze TH, Koller A, Kaley G (2005) Type 2 diabetic mice have increased arteriolar tone and blood pressure: enhanced release of COX-2-derived constrictor prostaglandins. Arterioscler Thromb Vasc Biol 25: 1610-1616

Fantin A, Vieira JM, Plein A, Maden CH, Ruhrberg C (2013) The embryonic mouse hindbrain as a qualitative and quantitative model for studying the molecular and cellular mechanisms of angiogenesis. Nature protocols 8: 418-429

Heiss M, Hellstrom M, Kalen M, May T, Weber H, Hecker M, Augustin HG, Korff T (2015) Endothelial cell spheroids as a versatile tool to study angiogenesis in vitro. FASEB journal : official publication of the Federation of American Societies for Experimental Biology 29: 3076-3084

Lanahan AA, Lech D, Dubrac A, Zhang J, Zhuang ZW, Eichmann A, Simons M (2014) PTP1b is a physiologic regulator of vascular endothelial growth factor signaling in endothelial cells. Circulation 130: 902-909

Limbourg A, Korff T, Napp LC, Schaper W, Drexler H, Limbourg FP (2009) Evaluation of postnatal arteriogenesis and angiogenesis in a mouse model of hind-limb ischemia. Nature protocols 4: 1737-1746

Lobov IB, Renard RA, Papadopoulos N, Gale NW, Thurston G, Yancopoulos GD, Wiegand SJ (2007) Delta-like ligand 4 (Dll4) is induced by VEGF as a negative regulator of angiogenic sprouting. Proceedings of the National Academy of Sciences of the United States of America 104: 3219-3224

Nakatsu MN, Davis J, Hughes CC (2007) Optimized fibrin gel bead assay for the study of angiogenesis. Journal of visualized experiments : JoVE: 186

Wang JM, Chen AF, Zhang K (2016) Isolation and Primary Culture of Mouse Aortic Endothelial Cells. Journal of visualized experiments : JoVE
