# Supplementary figures and images for: Intracellular adenosine regulates epigenetic programming in endothelial cells to promote angiogenesis
Source: EMBO Mol Med. 2017 Jul 27;9(9):1263–78. doi: 10.15252/emmm.201607066 (PMC5582416; doi:10.15252/emmm.201607066)

## Source Data for Appendix Figure S6

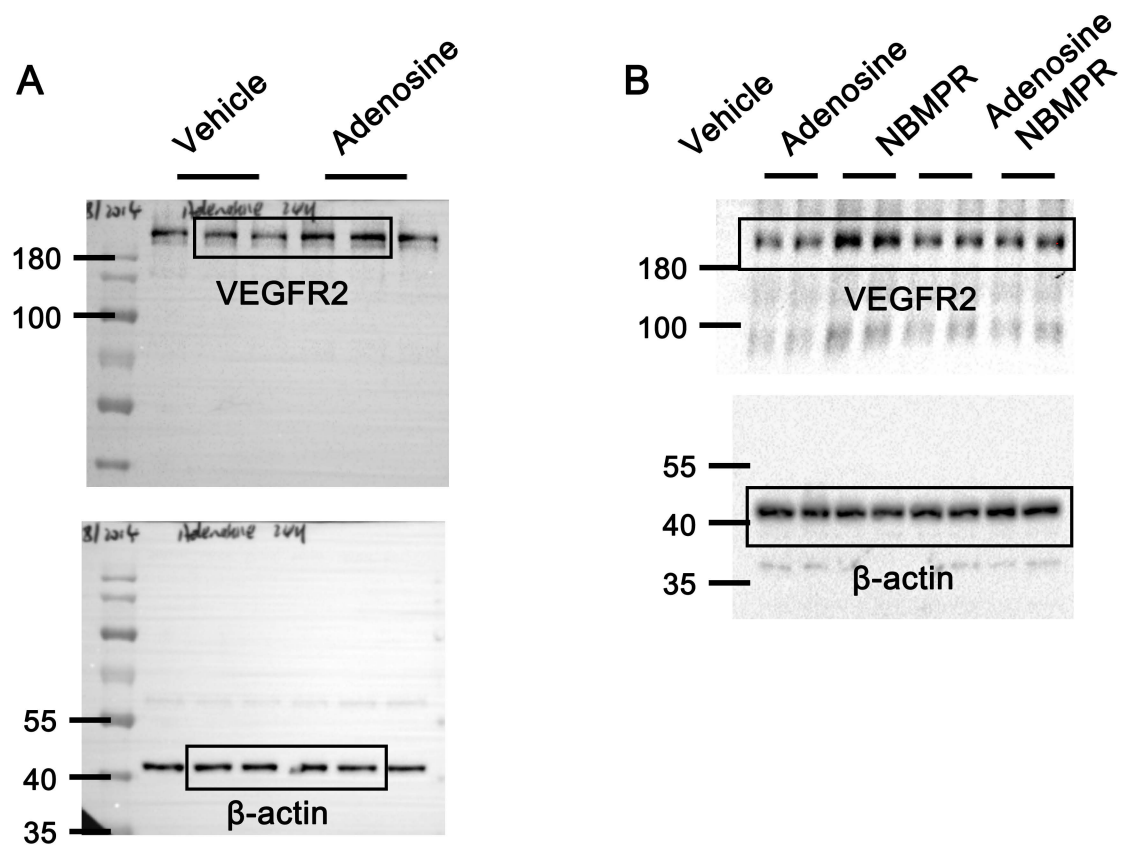

Supplement: Supplementary file 2 — Source Data for Appendix [file EMMM-9-1263-s008.zip › Source_Data_for_Appendix/Source_Data_for_Appendix_Figure_S6.pdf]

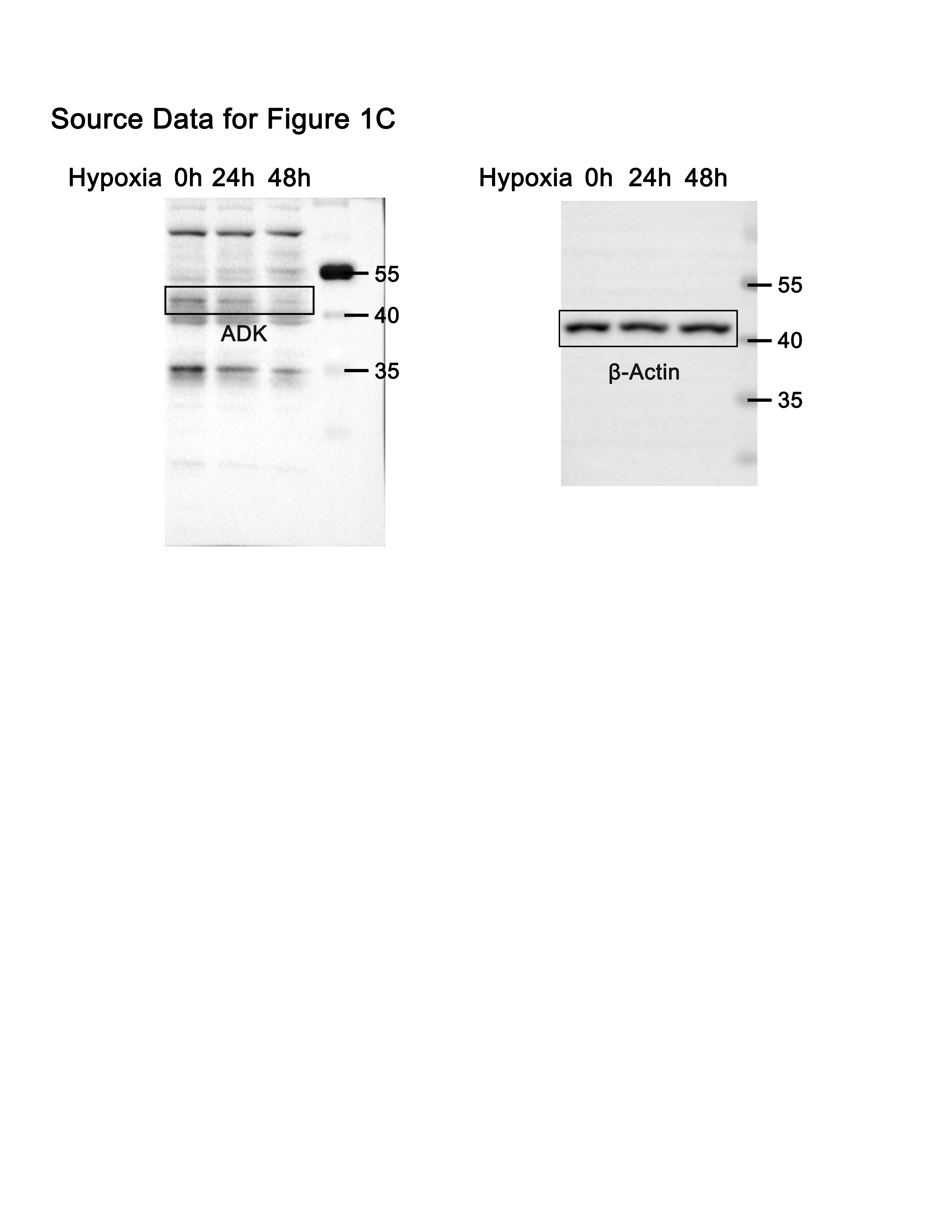

Supplement: Supplementary file 4 — Source Data for Figure 1 [file EMMM-9-1263-s002.tif]

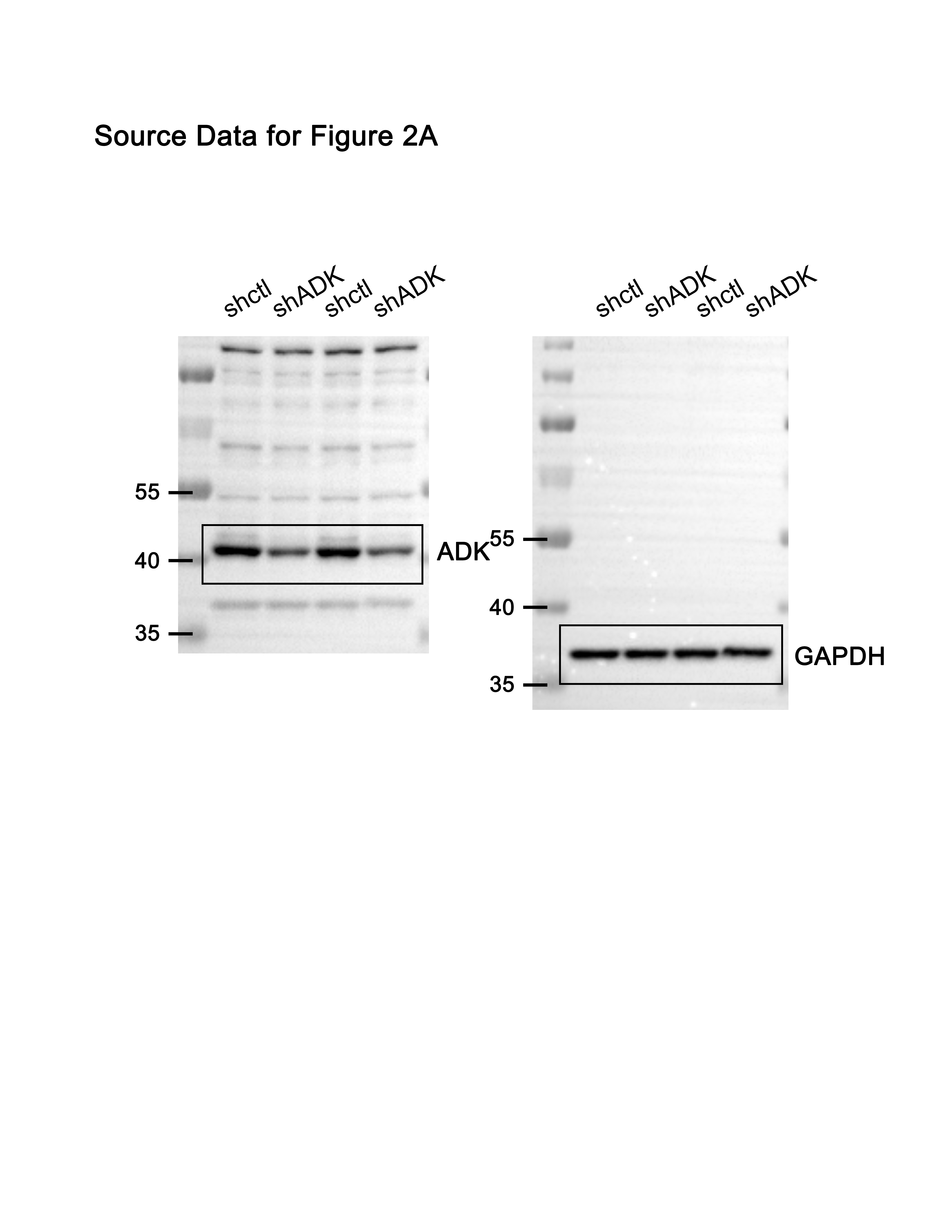

Supplement: Supplementary file 5 — Source Data for Figure 2 [file EMMM-9-1263-s003.tif]

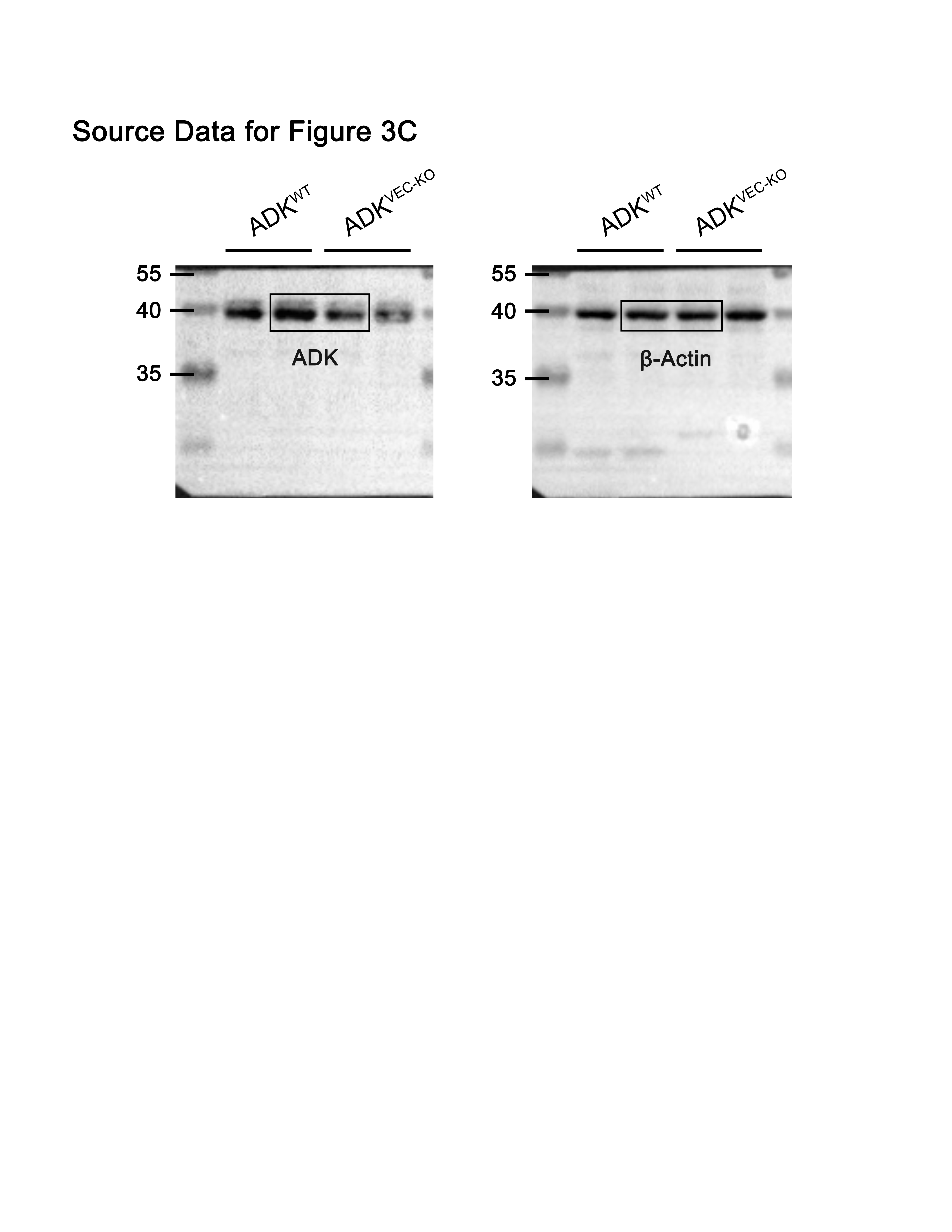

Supplement: Supplementary file 6 — Source Data for Figure 3 [file EMMM-9-1263-s004.tif]

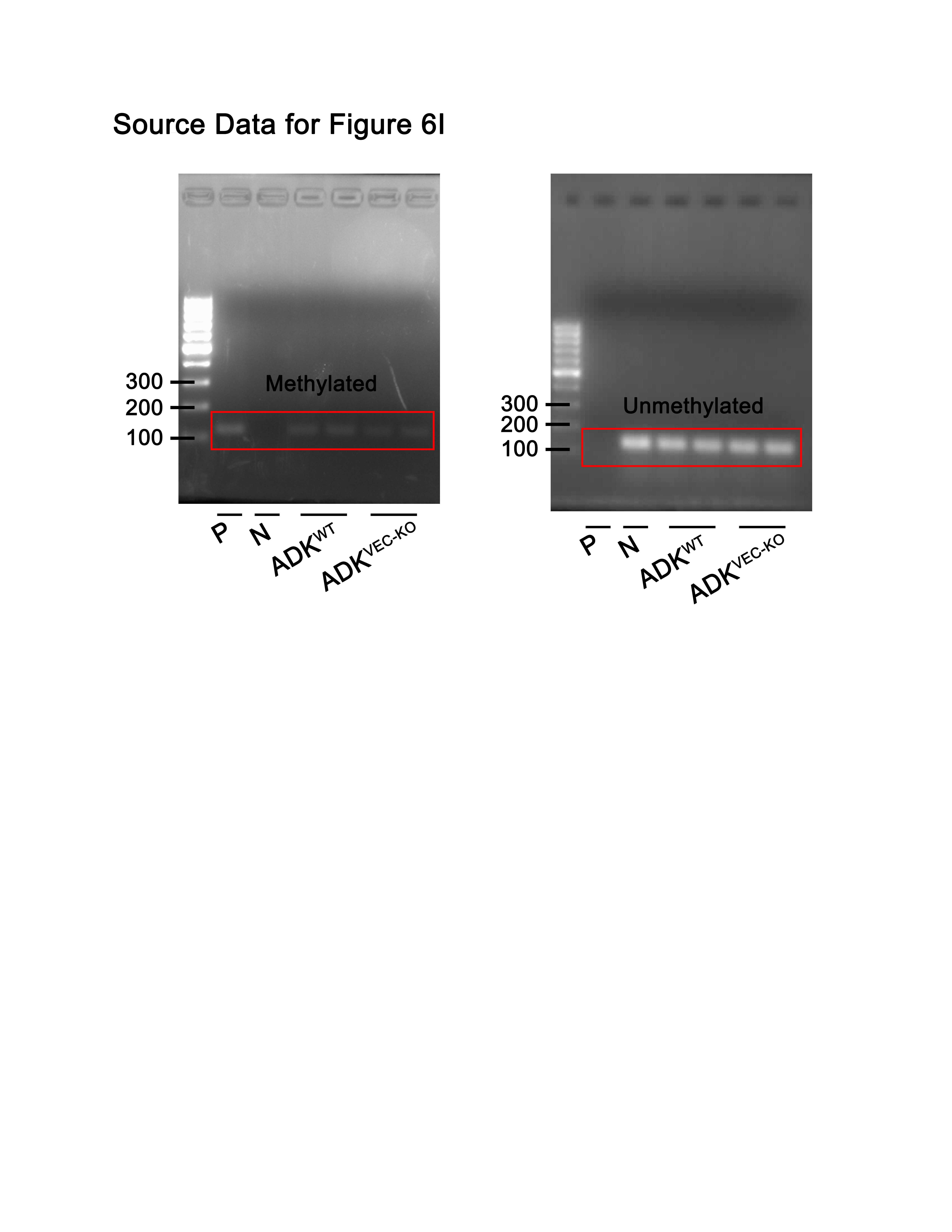

Supplement: Supplementary file 7 — Source Data for Figure 6 [file EMMM-9-1263-s005.tif]

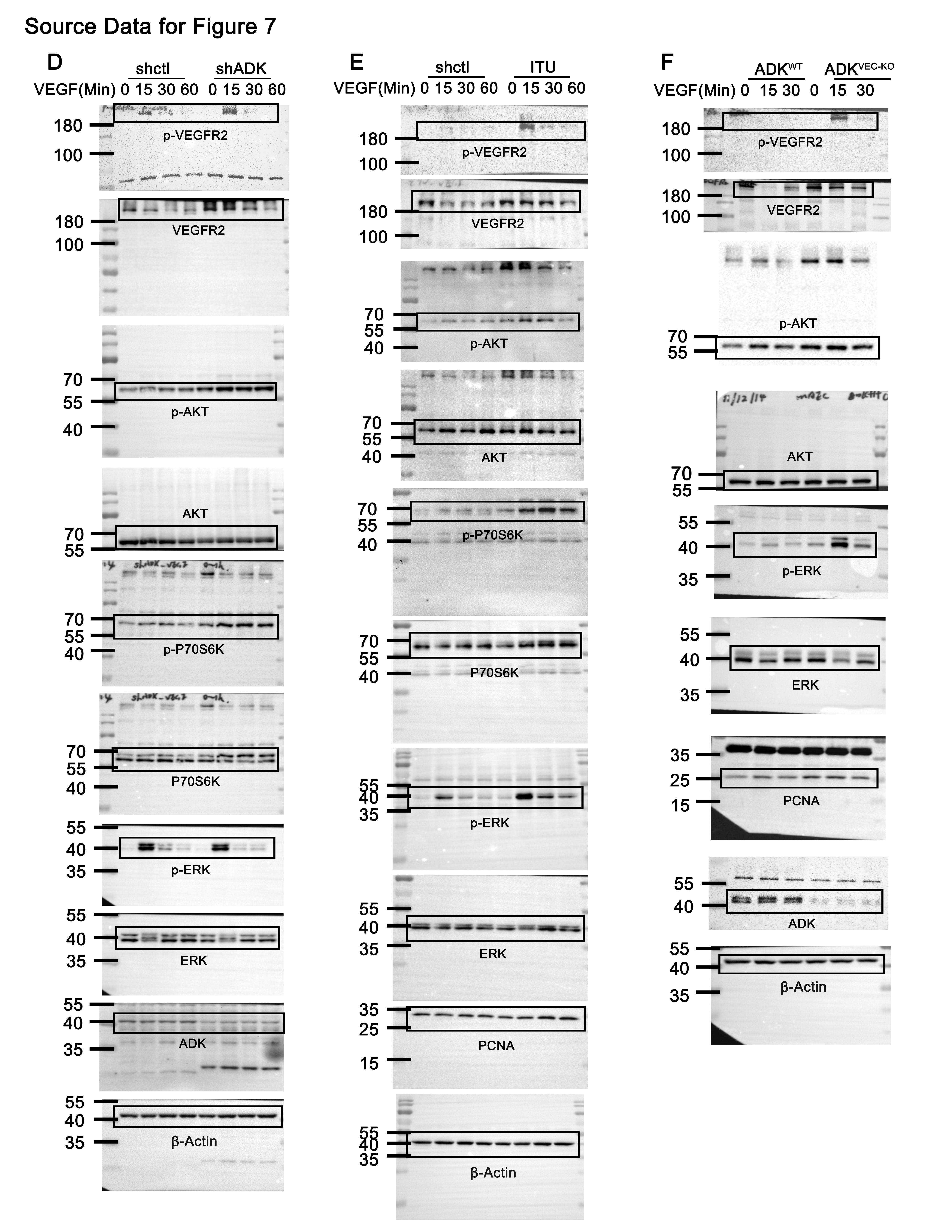

Supplement: Supplementary file 8 — Source Data for Figure 7 [file EMMM-9-1263-s006.tif]

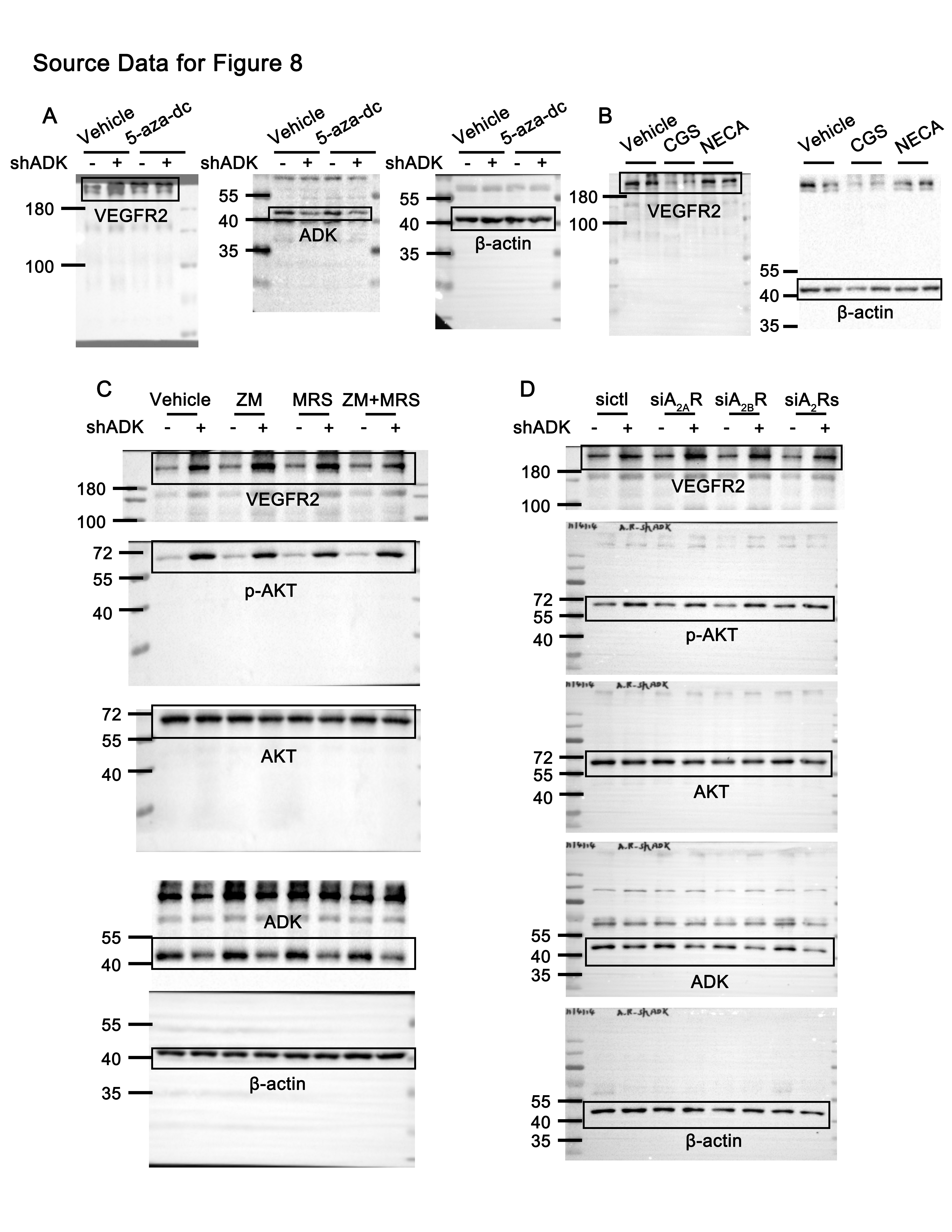

Supplement: Supplementary file 9 — Source Data for Figure 8 [file EMMM-9-1263-s007.tif]
